# The evolution of the Puf superfamily of proteins across the tree of eukaryotes

**DOI:** 10.1186/s12915-020-00814-3

**Published:** 2020-06-30

**Authors:** Vladimíra Najdrová, Courtney W. Stairs, Martina Vinopalová, Luboš Voleman, Pavel Doležal

**Affiliations:** 1 Department of Parasitology, Faculty of Science, Charles University, BIOCEV, Průmyslová 595, 252 50 Vestec, Czech Republic; 2 Department of Cell and Molecular Biology, Science for Life Laboratory, Uppsala University, SE-75123 Uppsala, Sweden

**Keywords:** RNA-binding protein, RNA processing, LECA, Puf superfamily proteins, *Giardia intestinalis*

## Abstract

**Background:**

Eukaryotic gene expression is controlled by a number of RNA-binding proteins (RBP), such as the proteins from the Puf (Pumilio and FBF) superfamily (PufSF). These proteins bind to RNA via multiple Puf repeat domains, each of which specifically recognizes a single RNA base. Recently, three diversified PufSF proteins have been described in model organisms, each of which is responsible for the maturation of ribosomal RNA or the translational regulation of mRNAs; however, less is known about the role of these proteins across eukaryotic diversity.

**Results:**

Here, we investigated the distribution and function of PufSF RBPs in the tree of eukaryotes. We determined that the following PufSF proteins are universally conserved across eukaryotes and can be broadly classified into three groups: (i) Nop9 orthologues, which participate in the nucleolar processing of immature 18S rRNA; (ii) ‘classical’ Pufs, which control the translation of mRNA; and (iii) PUM3 orthologues, which are involved in the maturation of 7S rRNA. In nearly all eukaryotes, the rRNA maturation proteins, Nop9 and PUM3, are retained as a single copy, while mRNA effectors (‘classical’ Pufs) underwent multiple lineage-specific expansions. We propose that the variation in number of ‘classical’ Pufs relates to the size of the transcriptome and thus the potential mRNA targets. We further distinguished full set of PufSF proteins in divergent metamonad *Giardia intestinalis* and initiated their cellular and biochemical characterization.

**Conclusions:**

Our data suggest that the last eukaryotic common ancestor (LECA) already contained all three types of PufSF proteins and that ‘classical’ Pufs then underwent lineage-specific expansions.

## Background

The Puf superfamily (PufSF) of proteins encompasses a class of eukaryotic RNA-binding proteins (RBPs), which interact with the 3′-untranslated regions (3′-UTRs) of mRNA in the cytosol or with the precursors of rRNA molecules in the nucleolus [1–3]. The name of the protein family is derived from Pumilio and Fem-3 binding factor of *Drosophila melanogaster* and *Caenorhabditis elegans*, respectively [4, 5]. PufSF proteins are defined by the presence of Puf repeats, each of which binds a single base of an RNA molecule [6–9]. In the past two decades, three types of PufSF proteins (i.e. Puf, Nop9, and PUM3, discussed below) have been distinguished according to their biological role and structural arrangement [2, 10–12].

The so-called classical Puf proteins (Pufs) bind 3′-UTRs of mRNA and usually have eight Puf repeats that organize into a crescent-shaped structure [13]. Each Puf repeat contains a tripartite recognition motif (TRM), and it is the combinations of eight TRMs, which specifies the sequence motif at which a particular Puf binds. In vivo, a single Puf protein can recognize hundreds of transcripts and, together with additional protein partners, regulate protein translation [1]. Puf proteins can mediate the repression [14–16], the activation [17] of gene expression, or the site-specific translation [18, 19]. These proteins function in the cytoplasm.

The two other types of PufSF proteins operate predominantly within the nucleolus, where ribosome biogenesis takes place [20]. Nop9 proteins carry 11 Puf repeats organized into a U-shaped structure, which participate in the processing and folding of 18S rRNA in the nucleolus [10, 21, 22], while PUM3 proteins (including the human PUM3/PufA and yeast Puf6) bind double-stranded DNA or RNA without a sequence specificity [2, 23]. Similar to Nop9, PUM3 proteins carry 11 Puf repeats, although organized in L-shaped domain, and they are involved in the nucleolar processing of the large subunit of the ribosome [23], potentially during the processing of 7S to 5.8S rRNA [2].

Although the PufSF proteins are essential for core processes of the eukaryotic cell, such as rRNA maturation, investigations of PufSF proteins have been restricted to only a few lineages of eukaryotes (e.g. Opisthokonta and Viridiplantae). These studies recognized that the number of PufSF proteins within and between lineages of eukaryotes is highly variable [24, 25]. Due to the number of different splice isoforms and the repetitive nature of the proteins, it has been difficult to delineate the evolutionary history of this essential protein family throughout eukaryotic evolution. Moreover, given the role of PufSF proteins in regulating the translation of a complex network of transcripts in modern eukaryotes [19, 26], it is tempting to speculate that PufSF proteins played a role in the origin of cellular complexity in eukaryotes. Indeed, such information is critical to understand the true nature of the last eukaryotic common ancestor (LECA); a concept of which can be drawn upon by examining the cellular properties of diverse eukaryotes, including the highly diverged organisms [27].

Metamonada represent highly diverged unicellular anaerobic eukaryotes [28] carrying specialized mitochondria [29]. Some Metamonada are important human and animal pathogens [30]. One of the best studied species of Metamonada is the human intestinal parasite *Giardia intestinalis*. Although the RNA metabolism in *G. intestinalis* is poorly understood, we would argue that this organism can be useful in studying various aspects of eukaryotic RNA biology owing to its transcriptome streamlining and overall extreme biology. For example, unlike most eukaryotes, the processing of rRNA and the actual nature of *G. intestinalis* nucleolus are still under debate [31]. Moreover, *G. intestinalis* generates large number of sterile transcripts of unknown function, which are both capped and polyadenylated [32]. To date, only six *cis*-spliced and two *trans*-spliced transcripts have been described in *G. intestinalis* making it easier to predict the transcriptome purely from genomic data [33, 34]. The 5′-untranslated regions (5′-UTRs) of *G. intestinalis* mRNAs are efficiently capped and bound by the ribosome despite being extremely short (i.e. 0–14 nucleotides) [35, 36]. Therefore, posttranscriptional regulation of gene expression is mostly limited to the stability and sequestration of the mRNAs [37]. Thus, 3′-UTRs remain the key regions of mRNAs, which affect its stability and localization via the interaction with RNA-binding proteins [37].

Here, we report systematic bioinformatic survey of distribution of PufSF proteins with sampling across major eukaryotic supergroups. Our analyses show three groups of proteins encompassing (i) Nop9, (ii) Puf, and (iii) PUM3 homologues. In a given organism, Nop9 and PUM3 are usually represented by a single gene, while the number of Pufs is highly variable. However, the actual number of Pufs correlates with the number of transcripts of the particular lineages and thus the number of putative mRNA targets. These data also suggest that the LECA already contained one Nop9, one PUM3, and two Puf proteins and that the large copy number of Pufs in modern organisms can be explain by lineage-specific expansions.

We were able to identify all three PufSF proteins even within *G. intestinalis.* However, their initial characterization points to unique adaptations in *G. intestinalis* RNA metabolism.

## Results

### Classification of PufSF proteins

**Fig. 1:**
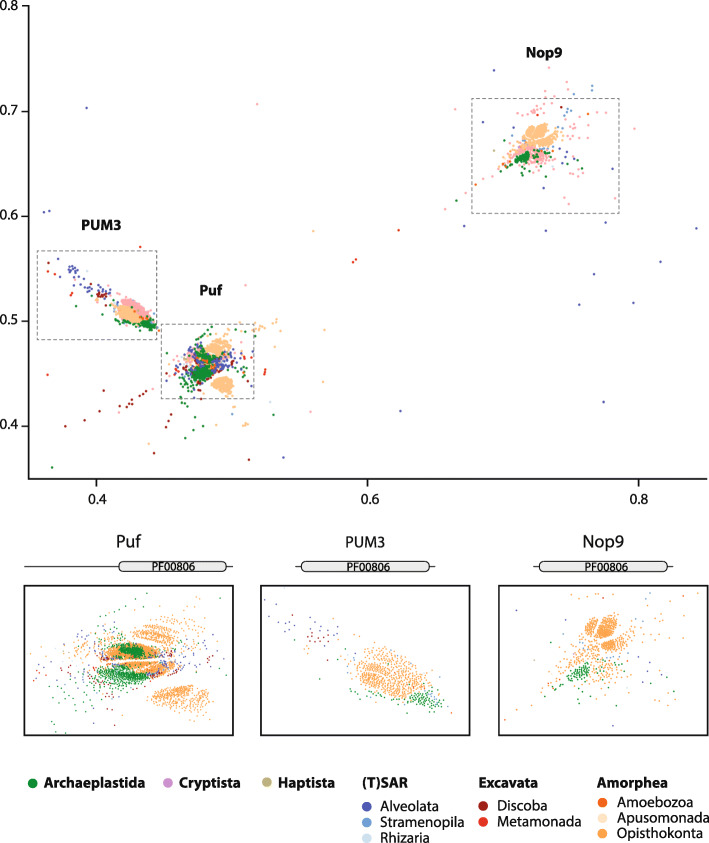
Clustering analysis of Puf family proteins. PufSF proteins were analysed by CLANS. A total of 4469 proteins formed three major clusters of proteins corresponding to Nop9 homologues (Nop9 cluster), ‘classical’ Puf proteins (Puf cluster), and PUM3 orthologues (PUM3 cluster). The protein sequences were colour-coded according to the taxonomic affiliation to the eukaryotic supergroup. The details of each large cluster show mostly lineage-specific subclusters, except two large subclusters of Puf proteins. The diagrams above the cluster details depict the general domain arrangement of PufSF proteins; PF00806 corresponds to Puf RNA-binding repeat. The *x* and *y* axes represent relative positions of the protein sequence in 2D CLANS

We initially classified the PufSF proteins across eukaryotic diversity. First, we performed a clustering analysis based on sequence similarity. This unbiased approach is based on mutual pairwise BLAST comparisons, and it is especially useful for the analysis of large protein datasets [38]. The initial dataset contained 4469 unique eukaryotic proteins carrying Puf repeat domain(s) (PF00806). No PufSF proteins were identified in Archaea or Bacteria, confirming the eukaryotic origin of the family. The clustering analysis revealed three major groups of PufSF proteins (Fig. 1, Additional file 1: Table S1): the Puf cluster, consisting of 2955 proteins containing eight Puf repeats in the C-terminal part of the protein; the PUM3 cluster, consisting of 674 PUM3 orthologues, including plant PUM24 proteins, with up to eleven Puf repeats; and the Nop9 cluster, consisting of 675 Nop9 proteins including the plant PUM23 proteins.

The proximity of the Puf and PUM3 proteins in our clustering analysis suggests that these proteins are more similar at the sequence level when compared to Nop9s, despite carrying different numbers of Puf repeats. Given the conservation of 11 Puf repeats in both the rRNA-binding proteins PUM3 and Nop9 proteins, we suspect that this was the ancestral domain arrangement of all members of the PufSF. Following adaptation to interaction with mRNA molecules, the Puf proteins adopted a domain arrangement of only eight Puf domains [2, 21, 22]. The clustering approach was not sensitive enough to reveal detailed taxon-specific grouping of PufSF proteins except for the most represented eukaryotic groups of animals, plants, and fungi. The only exception was the formation of two clear large subclusters within the Puf cluster (Fig. 1) containing proteins of mixed taxonomic affiliation, which indicated the existence of at least two different Puf orthologues in the last common ancestor of eukaryotes. The position of eukaryotic supergroups encompassing protist lineages was rather dispersed across the subclusters. Metamonad proteins including the *G. intestinalis* sequences represented one of the most diverge proteins of the family.

### Taxonomic distribution of PufSF proteins

**Fig. 2:**
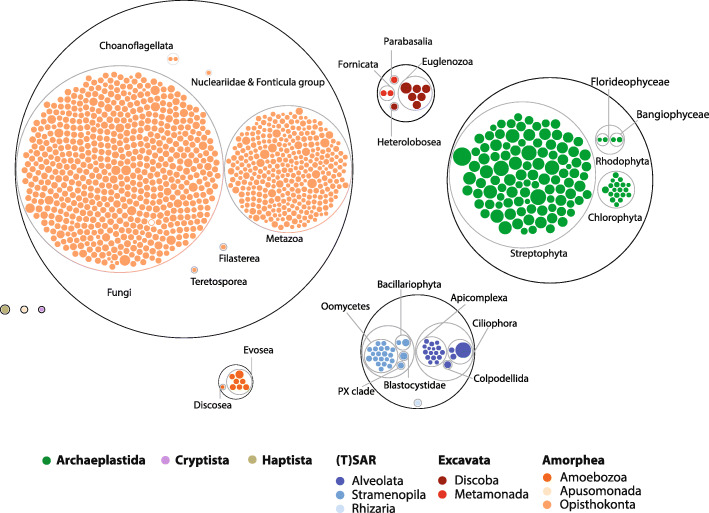
PufSF proteins in major groups of eukaryotes. Puf family proteins identified in all UniProt reference proteomes were grouped according to taxonomic affiliation as specified in Additional file 2: Table S2 and showed in circle packing plot. Each dot corresponds to a genome of particular eukaryotic species, and the size of the dot represents a number of Puf family proteins in the predicted proteome and the colour-codes to the taxonomic affiliation to the eukaryotic supergroup. Grey circles depict taxonomic groups

The number of PufSF proteins encoded in a given genome differs significantly across the eukaryotic diversity (e.g. [4, 25]). Hence, we surveyed the distribution of PufSF of proteins on a species level across the tree of eukaryotes. To this aim, we retrieved all eukaryotic 1180 reference proteomes from UniProtKB and classified them as Puf, PUM3, or Nop9 orthologues. While the dataset is biassed towards the proteomes from Opisthokonta and plants, it also contains curated proteomes of species from other eukaryotic supergroups. We used combination of InterPro precomputed protein families as a final determiner for the affiliation to one of the PufSF members (IPR001313—Puf repeat, IPR040000—Nop9, IPR040059—PUM3). In total, 7762 proteins were identified, and the proteins were classified according to hierarchic taxonomic groups (Fig. 2, Additional file 2: Table S2). Plotting the taxonomic distribution of the proteins showed that the highest number of PufSF proteins can be found in most of the plant species (Streptophyta), where the number of proteins ranged from 10 to 50. Extremely high number of proteins was also identified in ciliate *Paramecium tetraurelia* (43) but not in other ciliates or alveolates. In addition, all organism of analysed Euglenozoa group such as parasitic *Trypanosoma* and *Leishmania* species showed higher number of proteins (12–22). On the opposite end of the spectrum were parasites with reduced genomes, especially microsporidia or *Cryptosporidium* species with only one or two PufSF proteins. However, many animal taxa including insect and nematodes were also found to have only two or three proteins. Hence, at this point, we observed no clear relationship between the number of PufSF proteins and the biology or complexity of the surveyed organisms.

### Three types of PufSF proteins have undergone different evolution in eukaryotes

**Fig. 3:**
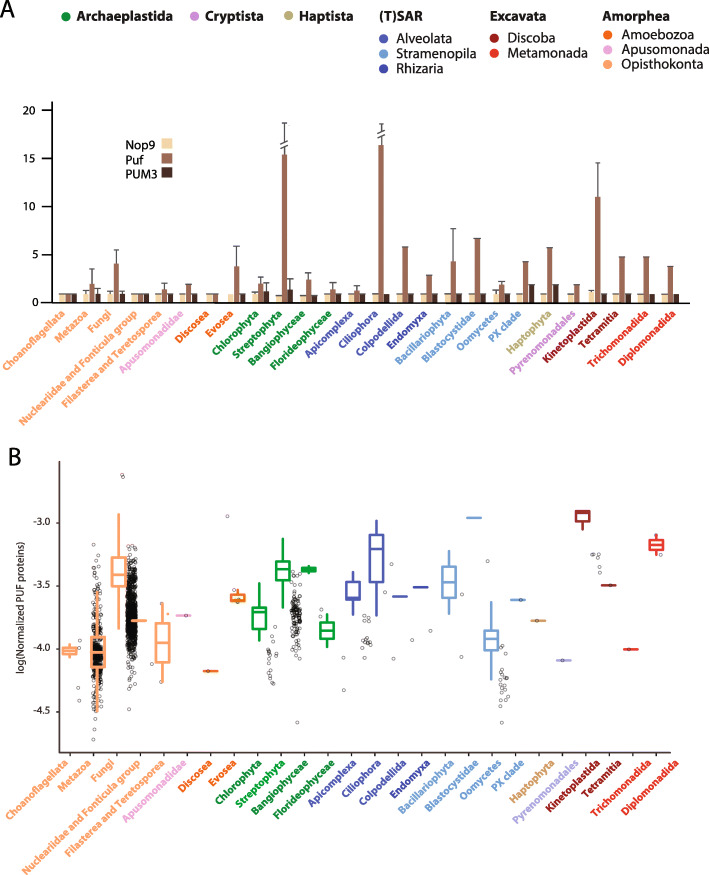
Three types of PufSF proteins in major groups of eukaryotes. **a** The number of Nop9, Puf, and PUM3 orthologues was identified for each proteome in the dataset, and the values were averaged for the taxonomic group of eukaryotes. The error bars correspond to the standard deviation of variance of values within the particular taxonomic group. Error bars for Streptophyta and Ciliophora were cut for better visualization. **b** The number of Pufs normalized with respect to the total number of the protein coding; colour-codes correspond to the taxonomic affiliation to the eukaryotic supergroups

Of all 7762 proteins from the reference proteomes, 1135 Nop9s, 5423 Pufs, and 1204 PUM3 proteins were identified (Additional file 2: Table S2). Interestingly, on average, each eukaryotic species contains a single Nop9 and PUM3 homologue and five Pufs (Fig. 3a). Moreover, while the number of Pufs is highly variable among lineages, the occurrence of single Nop9 and PUM3 proteins seems to be retained across eukaryotic diversity. The high number of PufSF proteins found in plants, Euglenozoa, and other species reflects lineage-specific amplification of the Puf proteins and not the ancestral state of early eukaryotes (Fig. 3a). The discrepancy observed between Nop9 and PUM3 gene copy number compared to Puf copy number might be related to different selective pressures experienced by these proteins owing to their role in the biogenesis of ribosomal RNA. On the other hand, given the Pufs’ role in controlling the translation of multiple mRNAs—which will vary between organisms—it is possible that the number of Pufs will differ and could instead relate to the total number of the protein-coding genes in the cell. In order to test this hypothesis, we normalized the number of Pufs with respect to the total number of the protein-coding genes in the corresponding species (Fig. 3b) (Additional file 2: Table S2). Interestingly, the resulting ratio between Pufs and the pool of putative target transcripts seem to be very similar across eukaryotes (Fig. 3b) with the average number of one Puf for every 3.47 × 10^4^ protein-coding genes.

### Phylogenetic reconstruction of PufSF proteins

**Fig. 4:**
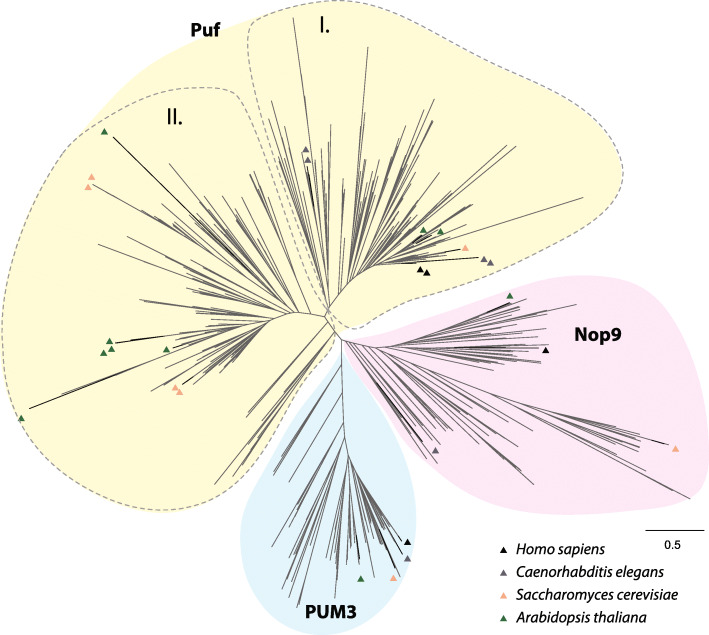
Phylogenetic analysis of PufSF proteins. Maximum likelihood phylogenetic inference of PufSF proteins with sequences from *H. sapiens*, *C. elegans*, *S. cerevisiae*, and *A. thaliana* is shown as triangles with the indicated colours. For visualization purposes, support values were removed. Full phylogenies of the PUM3, Nop9, and Puf proteins can be found in Additional file 3: Fig. S1

In order to get insight into the evolution of PufSF proteins, we performed the phylogenetic analyses on a dataset containing all three types of the proteins or the just a particular subset of either Puf, PUM3, or Nop9 orthologues (see the ‘Materials and methods’ section for more details). While the phylogenetic reconstructions proved to be problematic due to the repetitive structure of PufSF proteins, the overall tree (Fig. 4, Additional File 3: Fig. S1) shows three distinct clades corresponding to Puf, PUM3, and Nop9. Subsequent subtrees of PUM3 and Nop9 proteins, which are present as single proteins, resolved all major eukaryotic groups with some unexpected position of orthologues mainly from the Metamonada supergroup (Additional File 3: Fig. S1), most likely caused by their high sequence divergence.

Given the presence of several Pufs in most eukaryotes, we endeavoured to resolve their evolutionary relationships and specifically if the presence of multiple Pufs reflects the ancestral state of LECA or rather they are independent paralogues arisen by linage-specific gene duplication(s). The phylogenetic reconstructions of Pufs remained very problematic, and despite using distinct alignment strategies, they did not return clear separation among individual Pufs and the eukaryotic taxa (Additional File 3: Fig. S1). However, we could identify two clear groups (labelled ‘I’ and ‘II’) (Fig. 4) that encompass the vast majority of eukaryotic taxa. This possibly reflects the presence of only two Pufs in LECA. Within group I and group II, there have been a number of lineage-specific duplications giving rise to the multitude of Pufs seen in different genomes.

### PufSF proteins in the metamonad *G. intestinalis*

**Fig. 5:**
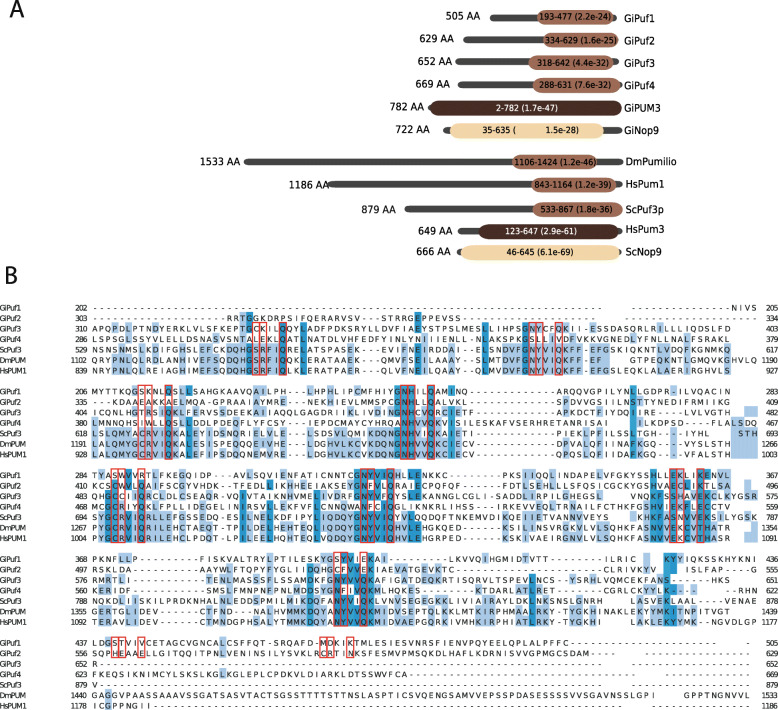
Domain structure of *G. intestinalis* PufSF proteins. **a** The Pumilio homology domain containing Puf repeats was predicted using HHPred against Pfam database and denoted as oval for each of the *Giardia intestinalis* proteins (shades of brown). The numbers in the ovals represent the position of the domain within the protein (black lines). The expectation value (*E* value) of the domain detection is shown in brackets. The fruit fly, human, and fungal orthologues are shown for comparison. Gi, *Giardia intestinalis*; Dm, *Drosophila melanogaster*; Hs, *Homo sapiens*; Sc, *Saccharomyces cerevisiae*. **b** Protein sequence alignment of *G. intestinalis* Pufs with selected proteins from *S. cerevisiae*, *D. melanogaster*, and *H. sapiens*. Open red rectangles highlight TRMs. Light and dark grey rectangles depict Puf repeats. Fraction of identical amino acids at the particular position is coloured: dark blue > 80%, blue > 60%, light blue > 40%, white < 40%

To test whether PufSF proteins are conserved in some of the most divergent eukaryotes, we specifically investigated the presence of PufSF proteins in *G. intestinalis*. Using a variety of sensitive sequence searching strategies (see the ‘Materials and methods’ section), we identified six proteins in *G. intestinalis.* The position and number of Puf repeats within the domain were predicted using HHpred and the alignments with the structurally characterized classical Pufs, Nop9, or PUM3 proteins, respectively. The classification of the proteins into the three types was confirmed by comparison with the domains defined at InterPro. Based on these classifications, we identified four *G. intestinalis* Puf homologues (GiPuf1–GiPuf4), one Nop9 (GiNop9), and PUM3 (GiPUM3) homologue (Fig. 5a). All four *G. intestinalis* Pufs were predicted to contain eight Puf repeats corresponding to eight TRMs (Fig. 5b), while both Nop9 and PUM3 homologues contained 11 Puf repeats similar to their homologues in *Saccharomyces cerevisiae* (Additional File 4: Fig. S2 and S3). The prediction of TRMs was performed by HHpred against the structurally characterized orthologues from *S. cerevisiae* and *D. melanogaster* (PDB ACNO. 5BZ1and 5KLA). However, the obtained TRMs for more divergent GiPuf1 and GiPuf2 were not in full agreement with the protein sequence alignment containing other *G. intestinalis* Puf proteins (Fig. 5b) as their Puf domain appeared shifted by two Puf repeats towards the C-terminus. At present, it is difficult to resolve if just the two Puf repeats were re-arranged or the entire domain was modified in these two proteins.

According to the phylogenetic reconstruction, the four *G. intestinalis* Pufs grouped together with the orthologues from closely related diplomonad species *Spironucleus salmonicida* and *Trepomonas* sp. distributed in group I and group II (Additional File 3: Fig. S1). GiPuf3, the most conserved Puf homologue of *G. intestinalis*, branched with group I Puf proteins while GiPuf1, GiPuf2, and GiPuf4 affiliated with the proteins from group II (Additional File 3: Fig. S1). The latter proteins thus likely represent lineage-specific gene duplications.

### Cellular localization of *G. intestinalis* PufSF proteins

In general, PufSF proteins localize to the nucleus or cytosol. In the cytosol, Puf proteins often associate with the cytoplasmic face of cellular compartments [1].

**Fig. 6:**
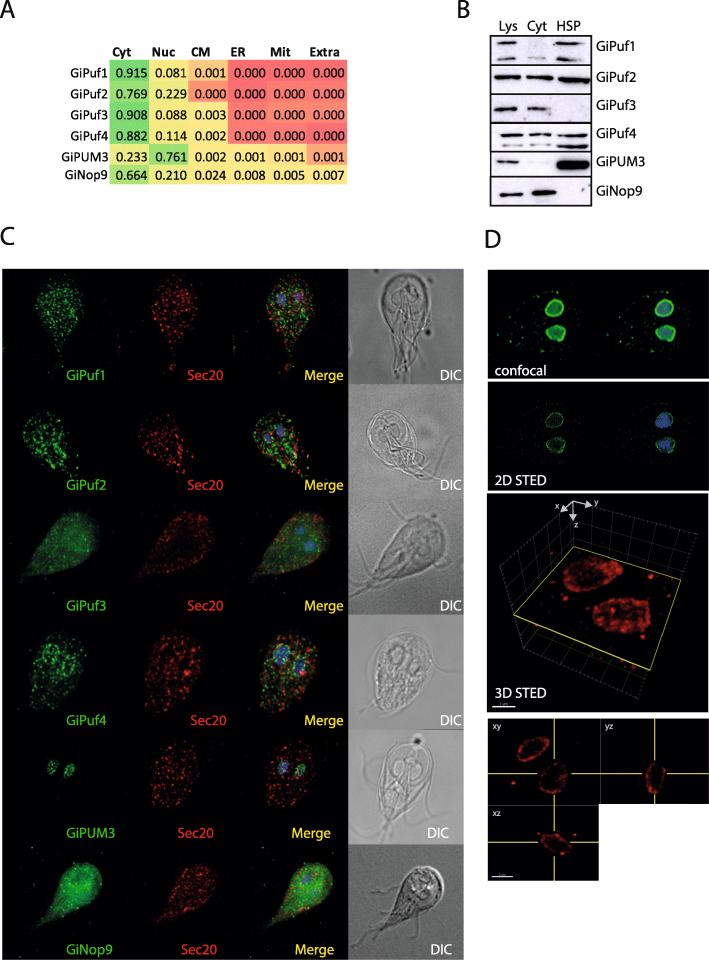
Cellular localization of *G. intestinalis* Puf and Nop9 proteins. **a** The scores obtained by DeepLoc prediction indicate cytosolic localization of all but GiPUM3 protein, which is predicted as nuclear protein. Colouring gradient represents the values from 0 (red) to 1 (green). Cyt, cytosol; Nuc, nucleus; CM, cytoplasmic membrane; ER, endoplasmic reticulum; Mit, mitochondrion; Extra, extracellular. **b** Western blot analysis of *G. intestinalis* expressing BAP-tagged PufSF proteins (one of at least three independent cell experiments is show). SDS-PAGE and immunoblots show total lysate (Lys), cytosolic (Cyt), and high-speed pellet (HSP) fraction. **c** Immunofluorescence analysis of the same cell lines shows cellular localization of the proteins. PufSF proteins in green, Sec20-endomembrane system marker in red. DIC, Differential Interference Contrast. **d** Detailed imaging of GiPUM3 by confocal and 2D STED (GiPUM3 in green, DNA in blue). 3D STED of GiPUM3 with the orthogonal projections (GiPUM3 in red)

To test the cellular localizations of each PufSF protein in *G. intestinalis*, we explored bioinformatic and experimental strategies. For bioinformatic predictions, we used DeepLoc [39], which uses neural networks to assess the localization of proteins based on a training set of experimentally localized proteins on UniProt. This algorithm predicted a cytoplasmic localization for all four *G. intestinalis* Pufs and Nop9 homologue and nuclear localization for only GiPUM3 (Fig. 6a). The cytosolic localization of the Pufs and the nuclear localization of GiPUM3 are in agreement with the expected roles of PufSF proteins, which control the stability and the localization of mRNAs in the cytosol and the nucleolar processing of 7S rRNA, respectively [2, 3]. However, given the role of Nop9 proteins in the maturation of pre-18S rRNA, the protein is expected to be in the nuclear compartment.

To test these subcellular localization predictions, we expressed all the *G. intestinalis* PufSF proteins with a C-terminal BAP (biotin acceptor peptide) tag in *G. intestinalis* and analysed their localizations with cell fractionation and microscopy using antibodies directed at the BAP tag. Expression of all constructs but one (GiPuf4) was highly unstable and diminished quickly after establishing stable cell lines. Therefore, *G. intestinalis* cell lysates were obtained as soon as possible and separated into two fractions: (i) the high-speed pellet, containing sedimentable membrane-bound organelles such as the nucleus, endoplasmic reticulum, peripheral vacuoles, or mitosomes, and (ii) the cytoplasmic fraction [40] (Fig. 6b). In general, the Puf proteins showed three different types of distribution: GiPuf3 and GiNop9 were found specifically in the cytosolic fraction, while GiPuf1 and GiPUM3 were present predominantly in high-speed pellet fraction. Finally, GiPuf2 and GiPuf4 showed the presence in both cytosolic and high-speed pellet fractions indicating their partial association with the cellular membranes.

In agreement with the western blot analyses, the immunofluorescence confocal microscopy of GiPuf3 and GiNop9 showed mainly cytosolic localization of the proteins with some punctate distribution in the cell that do not co-localize with the endomembrane marker Sec20 (Fig. 6c). While the data are in agreement with the bioinformatic prediction, the cytosolic presence of *G. intestinalis* Nop9 homologue remains puzzling. GiPuf1, GiPuf2, and GiPuf4 were present in different kinds of vesicular structures likely corresponding to specific regions of the endomembrane system, which however did not co-localize, with our endomembrane marker protein Sec20 (Fig. 6c). In addition, GiPuf2 and GiPuf4 showed also a perinuclear staining, which indicated that the protein is associated with the nuclear membrane. Conversely, a very specific labelling of two *G. intestinalis* nuclei was observed for GiPUM3. In other eukaryotes, PUM3 localizes to discrete nucleolar spots in the nuclear matrix [3, 10]. To determine the subnuclear localization of GiPUM3, we performed high-resolution STED microscopy. In both 2D and 3D STED microscopy, we observed GiPUM3 localizing to the periphery of the nucleus (Fig. 6d).

To determine potential Puf-interacting proteins in *G. intestinalis*, we explored the Puf-interactome using a high-resolution proximity labelling coupled to mass spectrometry. By determining potential interaction partners of the *G. intestinalis* Pufs, we could better predict their involvement in gene expression control. Unfortunately, the expression of tagged Pufs was highly unstable and diminished quickly after the cell transformation and we thus could not perform larger scale experiments required for protein- or RNA-pull down experiments. We were, however, able to generate a cell line weakly expressing BAP-tagged GiPuf4 in the presence of cytosolic biotin ligase BirA [41]. Upon crosslinking and purification of GiPuf4 on streptavidin-coupled Dynabeads, the triplicate samples were analysed by mass spectrometry. The purified GiPuf4 was found to be specifically enriched in our sample, although the experiment did not reveal any specific interacting partner protein above the statistical threshold (Additional file 5: Fig. S4, Additional file 6: Table S3). Thus, any functional predictions could not be drawn at this stage.

### Prediction of binding motifs of *G. intestinalis* Pufs and their target mRNA

**Fig. 7:**
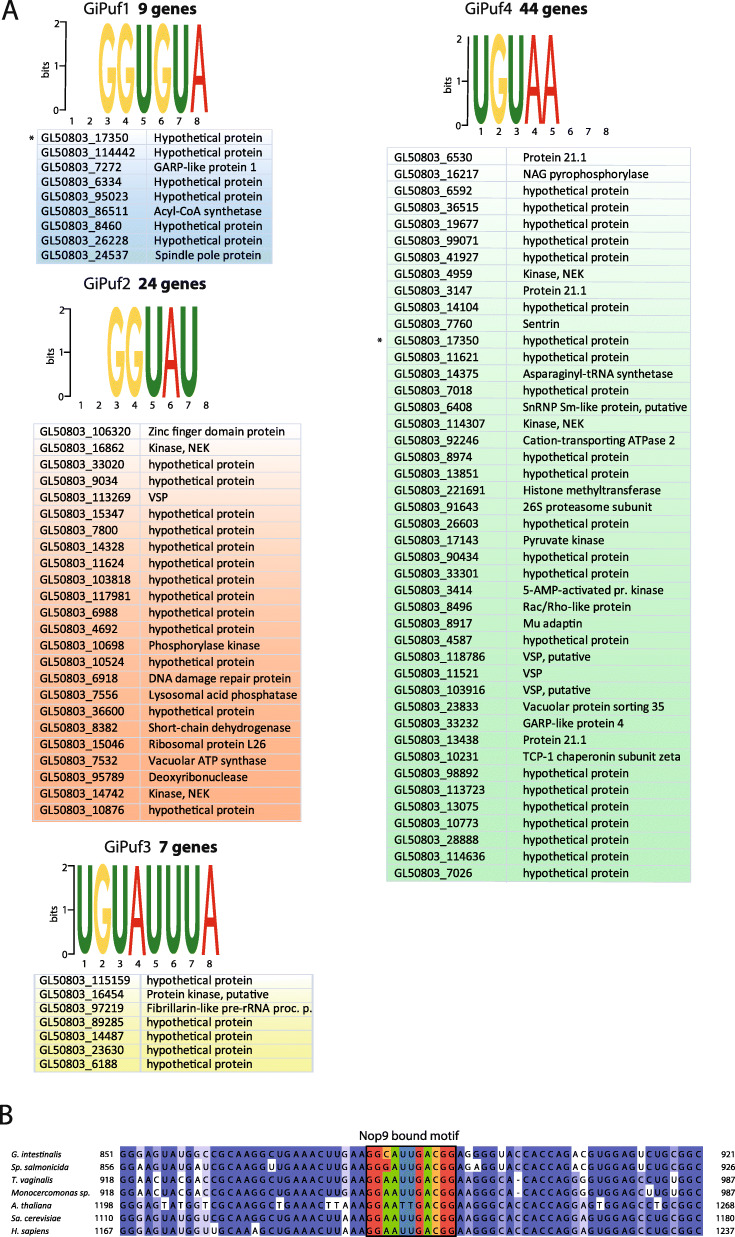
Predicted binding motifs of *G.intestinalis* PufSF proteins. **a** The tripartite recognition motifs (TRMs) of each Puf repeats of *G.intestinalis* Pufs were predicted, and the resulting sequence was used to search the conceptual transcriptome. The number of putative mRNA targets, which contain the motif in the 3′-UTR, is shown in bold. Asterisk denotes the same putative mRNA recognized by two different Pufs. **b** Sequence alignment of 18S rRNA shows the conservation of the sequence recognized by Nop9 as it was experimentally identified for *S. cerevisae* Nop9. Fraction of identical nucleotides at the particular position is coloured: dark blue > 80%, blue > 60%, light blue > 40%, white < 40%

While we could not identify the interacting factors for *G. intestinalis* Pufs by mass spectrometry, we decided to predict the sets of recognized mRNAs for each homologue. The RNA sequence motif recognized by Puf/Nop9 proteins is determined by the combination of three amino acid residues, referred to as tripartite recognition motif (TRM). TRM is part of five residues in the second α-helix of each Puf repeat represented as 1-2-X-X-5 (where X is any hydrophobic residues). Within the TRM, positions 1 and 5 bind the edge of the RNA base, while the position 2 makes a stacking interaction with RNA molecule [42]. Some TRMs have been shown to be specific for particular base [11]. Hence, upon the identification of the TRMs in each Puf repeat, it is possible to predict its sequence-specific binding properties [9]. However, it should be noted that for some naturally occurring TRMs, the specificity has not been determined. By comparing the most closely related sequences to each *G. intestinalis* Puf identified with HHpred, we predicted the putative binding motif by manually checking the position of individual Puf repeats (Fig. 5b), and we could predict putative RNA-binding motifs for all *G. intestinalis* Pufs (Fig. 7a). Several predicted TRMs located in GiPuf1, GiPuf2, and GiPuf4 contained experimentally unidentified amino acid combinations which left these putative binding motifs incomplete. Interestingly, a complete binding motif predicted for GiPuf3 (5′-UGUAUUUA-3′) was found to be highly similar to 5′-UGUAUAUA-3′motif of prototypical members of the protein family such as human PUM1 or yeast Puf3 [43].

The predicted motifs of *G. intestinalis* Pufs were then used to search the dataset of theoretical 3′-UTR of all 9747 *G. intestinalis* genes retrieved from GiardiaDB. Given that the 3′-UTRs of *G. intestinalis* mRNAs are very short [30, 32, 44], the length of the UTRs was limited to 50 bases only.

Using the FIMO (Find Individual Motif Occurrence) algorithm [45], a specific set of possible cognate mRNAs for GiPuf1–GiPuf4 was retrieved (Fig. 7, Additional file 8: Table S5). Each of the *G. intestinalis* Puf proteins was predicted to interact with the different number of transcripts, and this number was also inversely proportional to the *G. intestinalis* length of the predicted binding motif: where GiPuf3, GiPuf1, GiPuf2, and GiPuf4 were predicted to interact with 7, 9, 24, and 44 transcripts, respectively. These numbers are substantially lower than other Puf proteins that are predicted to interact with hundreds of RNA targets [19, 46]. We next explored the putative function and subcellular localization of the protein products of the predicted target transcripts (Additional file 8: Table S5). Interestingly, the target transcripts included other RNA-processing proteins (e.g. fibrillarin) and a component of the ERAD (endoplasmic reticulum-associated degradation) pathway (e.g. Derlin 1) (Additional file 8: Table S5).

Unlike Pufs, Nop9s use their 11 Puf repeats [21, 22] to specifically bind to the pre-18S rRNA at the central pseudoknot region [22] and regulate its processing possibly by competing with Nob1 nuclease [21]. However, how Nop9 TRMs interact with the rRNA was unknown until recently where it was shown that yeast Nop9 binds to a specific 11-nucleotide region of the pre-18S rRNA [22]. The alignment of 18S rRNA sequences showed that this region is very well conserved across eukaryotes including species from the Metamonada group (Fig. 6b) suggesting that Nop9 might also recognize this region. However, in *G. intestinalis*, the cytoplasmic (and not nuclear) localization of this protein challenges whether GiNop9 and rRNA processing occurs in the nucleus.

Finally, when GiPUM3 was aligned with its characterized human and yeast counterparts, no previously identified TRMs could be identified within the sequence. This supported the absence of recognizable binding motif for this type of PufSF proteins (Additional File 4: Fig. S3) [2, 23].

## Discussion

RBPs stand at the centre of the regulation of eukaryotic protein expression by mediating a wide range of posttranscriptional processes. There are several distinctive molecular properties of PufSF proteins [4, 5]. The protein structure is composed of 8–11 units (Puf repeats), combination of which defines the sequence specificity towards their RNA targets. They have been shown to provide molecular basis for long sought processes such as site-specific translation of proteins, RNA degradation, or maturation of rRNA [1].

Here, we explored the evolution of PufSF proteins across the tree of eukaryotes. In addition to the originally characterized ‘classical’ Pufs [4, 5], which mediate the turnover of mRNAs via binding to their 3′-UTRs [19], recent studies in yeast model system have defined two additional types of PufSF proteins, which perform distinct cellular functions. Nop9 binds pre-18S rRNA during its maturation [3, 21, 22], and PUM3 orthologues are involved in processing 7S rRNA [2, 10, 47] and positioning of specific mRNAs [48].

Given that PufSF proteins are involved in the core processes of the eukaryotic cell (e.g. the maturation of rRNA and the translational control of mRNAs), we hypothesized that this system should be conserved across eukaryotic diversity. Indeed, we found that all majority lineages of eukaryotes encode at least one homologue of Nop9, PUM3, and two Puf proteins (Figs. 2, 3, and 4; Additional file 2: Table S2). The lack of PufSF proteins in prokaryotic genomes (including the closest prokaryotic relatives of eukaryotes—the Asgard archaea [49]) suggests that these proteins are in fact eukaryotic innovations. Moreover, their ubiquity across the tree of eukaryotes (including the highly diverged metamonads) suggests they were already present in protoeukaryotes before the emergence of LECA. To uncover the within protein family evolutionary history of the PufSF proteins, we performed a clustering analysis on the basis of sequence similarity. We found that PufSF proteins form three larger groups corresponding to the three independent biological functions performed by Puf, PUM3, and Nop9 proteins.

This suggests that the Puf and PUM3 proteins likely originated from the same ancestral gene. Interestingly, however, Nop9 and PUM3 proteins, despite belonging to separate groups of proteins within the family, share an array of 11 Puf repeats by which they interact with pre-18S [21] or 7S rRNA [2], respectively. On the basis of clustering analysis, it seems either that both Nop9 and PUM3 independently evolved into 11 Puf repeat containing proteins or that Pufs evolved from a larger 11-repeat domain ancestral protein into an 8-repeat domain protein by disposing of three Puf repeats. The latter seems to be more plausible as Pufs acquired additional long N-terminal part of mostly unknown function, which may, however, carry additional RNA-binding motifs [50].

As discussed above, it is most likely that the Nop9, PUM3, and two Puf proteins evolved during eukaryogenesis prior to the emergence of LECA, which was already indicated by the existence of four eukaryotic orthologous groups (KOGs) specific for PufSF [51]. The role of PufSF proteins in both nuclear and cytoplasmic processes suggests that this protein family likely coincided with the evolution of a nucleic acid compartment; however, the exact timing cannot be determined with current data. Indeed, as protoeukaryotes began to complexify, it is tempting to speculate that functional networks necessary to manage an expanding genome and gene repertoire would demand additional levels of regulation—a role potentially filled by RBPs such as the PufSF proteins. Future examination of the relative timing [52] of the emergence of PufSF proteins and their experimental characterization in diverse eukaryotic lineages will be critical in assessing these hypotheses.

The classification of the protein family members also revealed that while the number of Pufs is highly variable across lineages, both Nop9 and PUM3 orthologues remained present as single copy genes with no paralogous sequences in the majority of eukaryotes. This can likely be attributed to the conserved role of the Nop9 and PUM3 proteins in the maturation of rRNA which is essential for the biogenesis of eukaryotic ribosome. Our analysis also demonstrated that the expansion of Pufs in some lineages of eukaryotes is similar to those documented before for *Arabidopsis thaliana* [25]. Interestingly, we showed that the actual number of Pufs in eukaryotic supergroups corresponds to the size of the proteome and therefore also the number of putative target mRNAs within the cell. Importantly, our estimates are based on the total number of genes present in a genome and will therefore be an underestimation of the total number of transcripts present in a cell resulting in an overestimated Puf to total gene ratio depicted in Fig. 3. However, in organisms such as *G. intestinalis* or yeast, which are intron-poor and lack methods for generating splice variants, the total number of transcripts is approximately equal to the total number of protein-coding genes. Taking this into account, the Puf to total gene ratio of *G. intestinalis* is one of the highest across eukaryotic diversity. This suggests that while the total number of transcripts might still be determinative of Puf number in other organisms, additional factors beyond transcript number seem to govern the persistence of multiple Pufs in *G. intestinalis*.

The unique cellular and molecular features of *G. intestinalis* and its specialization make it an ideal model to study the extreme limits of cell biology [53]. With respect to RNA metabolism, *G. intestinalis* is often the exception to the rule. Previous studies have demonstrated that *G. intestinalis* lacks core regulatory components of transcription [37] and has even repurposed some conserved RNA metabolic pathways [54]. Our analyses of PufSF proteins from *G. intestinalis* have added at least one piece to the puzzle of RNA metabolism where we were able to show that like other eukaryotes, *G. intestinalis* has retained genes encoding all the three core RBPs.

However, the initial characterization of the proteins has uncovered interesting deviations from the eukaryotic blueprint. *G. intestinalis* PUM3 orthologue (GiPUM3) is localized on the nuclear periphery, which is different from nucleolar localization found in all experimentally analysed organisms [10, 48, 55]. Similarly, the cytosolic localization of *G. intestinalis* Nop9 orthologue contradicts its expected role in pre-18S rRNA processing occurring in the nucleolus.

This raises a question on the nature and localization of *G. intestinalis* nucleolus. For long time, *G. intestinalis* has been thought to lack nucleolus-like structures since nucleolar markers have failed to show discrete nuclear labelling [56]. However, later hybridization of 18S rRNA and localization of fibrillarin, pseudouridine synthase, and snoRNA suggested specific distribution of the nucleoli on the periphery of both *G. intestinalis* nuclei [57–60]. It is therefore likely that the nuclear periphery functionally replaces nucleolar bodies typical of other eukaryotes [31].

Unexpectedly, we observed that GiNop9 localizes to the cytosol of *G. intestinalis*. We have a number of hypotheses that could explain this observation. First, we cannot rule out that the overexpression of the protein resulted in mistargeting of *G. intestinalis* Nop9, although we should be able to observe at least partial nuclear signal. Alternatively, GiNop9 does not participate in pre-18S rRNA processing in *G. intestinalis* or its cytosolic presence is a consequence of unique nucleolar localization in *G. intestinalis*. Nop9 is thought to prevent rRNA processing by cytosolic Nob1 nuclease by competing for binding at the same rRNA sequence [21]. Nob1 mediates the late step of pre-rRNA processing which generates 18S rRNA from 20S intermediate rRNA [61], and these interactions thus occur at the interface between the nucleus and cytosol. It is possible that in *G. intestinalis*, the relocation of the nucleolus to the nuclear periphery also relocated the latter steps of rRNA processing outside the nucleus whereby GiNop9 participates in the cytosolic maturation of the ribosome.

The bioinformatic survey for putative target mRNAs, whose 3′-UTR are bound by one of four *G. intestinalis* Pufs, returned only a small set of candidate genes compared to other eukaryotes [8]. This might stem from the extremely short UTRs in *G. intestinalis* [32, 37], which limits the actual region of interaction with RBPs. We could only confidently predict a complete binding motif (5′- UGUAUUUA-3′) for GiPuf3, and in this case, the motif was found to be highly similar to binding motifs of Pufs from other lineages of eukaryotes. The conservation of this binding motif in *G. intestinalis* was surprising given that *G. intestinalis* usually lacks many other core regulatory elements when compared to model eukaryotes (e.g. TATA box or Inr element) [62, 63]. Given the theoretical capabilities of Pufs to bind any possible RNA sequence [64], it raises an important question on the functional advantage of this particular binding motif in RNA-protein interactions.

Obviously, there are many unknowns left concerning the function of PufSF proteins especially in non-model organisms like *G. intestinalis* and other protists. However, despite numerous differences in posttransriptional processes found among eukaryotes, this work shows that PufSF proteins have constituted key RNA-processing proteins since LECA.

## Conclusions

In this study, we show that all three types of PufSF proteins are found across eukaryotic diversity. Moreover, we found that their role in both rRNA maturation (Nop9, PUM3) and mRNA translational control (Pufs) is conserved in all major lineages of eukaryotes and that they were present in the last eukaryotic common ancestor (LECA). Finally, we experimentally show the presence of three types of PufSF family proteins in a Metamonad *G. intestinalis* and highlight their several intriguing lineage-specific adaptations.

## Materials and methods

### Bioinformatic analyses

For the clustering analyses, the entry dataset of PufSF proteins was obtained from InterPro/UniProt database. All duplicates and sequences shorter than 500 amino acids were filtered out. Clustering analysis with CLANS was performed using MPI Bioinformatics toolkit available at https://toolkit.tuebingen.mpg.de/ [38]. The resulting 2D coordinates were plotted in GraphPad, and the dots representing the protein sequences were colour-coded according to current taxonomic descriptors of eukaryotes [65]. To identify putative Puf homologues in *G. intestinalis*, the protein alignment of PufSF was used in HHpred search (available at https://toolkit.tuebingen.mpg.de/#/tools/hhpred) against conceptual *G. intestinalis* proteome (Giardia_lamblia_ATCC_50803_31_Aug_2017) [66]. For the detection of Puf-binding motifs in 3′-UTRs, the motifs were generated by MEME (Multiple Em for Motif Elicitation available at http://meme-suite.org/tools/meme) [67] and used by FIMO (Find Individual Motif Occurrences) program (available at http://meme-suite.org/tools/fimo) [45] against our custom database of all conceptual 50-nt-long 3′-UTRs (obtained from GiardiaDB http://giardiadb.org/giardiadb/) [68]. Dataset of 18S rRNA was obtained from the high-quality ribosomal RNA database SILVA (available at https://www.arb-silva.de/) [69]. Sequences were aligned using mafft –auto [70], and alignment was coloured according to percentage identity in Jalview [71]. The proteins co-purified with GiPuf4 and identified by mass spectrometry were analysed by HHpred for homology detection and by DeepLoc (available at http://www.cbs.dtu.dk/services/DeepLoc/) for subcellular localization [39].

### Classification of PufSF proteins in eukaryotes

To assess the distribution of Nop9, PUM3, and Puf sequences across the tree of eukaryotes, we used the precomputed domain annotation on InterProScan (ftp://ftp.ebi.ac.uk/pub/databases/interpro/protein2ipr.dat.gz) of all eukaryotes present in the Reference Proteome database from UniProtKB. Importantly, since we were interested in understanding how many gene expansions occurred in each genome, we ignored splice variants by selecting only one representative protein per variant. Proteins were classified based on the presence of the following IPR domains/PANTHER family/SMART domain: Nop9 (IPR040000/PTHR13102), PUM3 (IPR040059/ PTHR13389), and Puf (IPR001313/SM00025).

### Phylogenetic dataset construction and analyses

For phylogenetic dataset construction, a subset of representative taxa across eukaryotic diversity were selected from the entry dataset from the clustering analysis. Due to the variety of different protein lengths, only the region corresponding to the Puf repeats was analysed; for each sequences, we extracted the region corresponding to the start position of the first, until the last position of the final, Puf repeat using HMMsearch with the SMRT profile (SM00025). Sequences were aligned using mafft-linsi [70], and ambiguously aligned sites were removed using trimal with the ‘-gappyout’ option [72]. Initial phylogenetic tree of the entire PufSF (i.e. Puf, Nop9, and PUM3 proteins) was generated using FastTree with the -lg option [73]. This tree was manually examined, and in the cases where an organism had multiple copies of a paralogue that were monophyletic, only one sequence was retained. This refined dataset was realigned as above. Maximum likelihood inference of the reduced dataset was performed using IQ-TREE v 2.0 [74] using the LG + G + F model. Given the large size of the dataset, we did not perform model testing with the mixture models for the PufSF tree. A total of 1000 SH-aLRTs (SH-like approximate likelihood ratio test; -alrt 1000), 1000 ultrafast bootstraps (-bb 1000) [75], 100 non-parametric boostraps (-b 100), and the transfer bootstrap expectation calculation (--tbe) [76] were performed and mapped onto the best-scoring maximum likelihood tree. To refine the phylogeny of Nop9, PUM3, and Puf proteins, we analysed each subclade separately. The sequences were aligned as above. Maximum likelihood inference of the reduced dataset was performed using IQ-TREE v 2.0 [74] under the best-scoring model of evolution selected by ModelFinder supplemented with the C-series mixture models using the -mset option [77]. A total of 1000 SH-aLRTs (SH-like approximate likelihood ratio test; -alrt 1000), 1000 ultrafast bootstraps (-bb 1000) [75], were mapped onto the best-scoring maximum likelihood tree. The resulting maximum likelihood tree was used as the guide tree (-ft) for rapid approximation of posterior mean site frequency (PMSF) of the C-series of mixture models [78] to generate 100 non-parametric bootstraps (-b 100) together with the transfer bootstrap expectation (--tbe). Alignment features and model parameters are specified in Additional file 7: Table S4, and datasets are available on the figshare repository (DOI:10.6084/m9.figshare.12097692), see ‘Availability of Data and Materials’.

### Cell culture, cloning, and transfection

The *G. intestinalis* strain WB (ATCC 30957) was grown in TYI-S-33 medium [79] supplemented with 10% heat-inactivated bovine serum, 0.1% bovine bile, and antibiotics at 37 °C. The genes encoding *G. intestinalis* PufSF proteins [GiardiaDB accession numbers GL50803_17262 (GiPuf1), GL50803_17590 (GiPuf2), GL50803_17325 (GiPuf3), GL50803_4548 (GiPuf4), GL50803_16602 (GiPUM3), and GL50803_14117 (GiNop9)] and 150-bp-long 5′-UTR of GiPuf1, GiPuf2, GiPuf3, GiPuf4, and GiNop9 were amplified from genomic DNA using PCR and inserted to the plasmid pOndra [40] with the C-terminal biotin acceptor peptide (BAP). Additional file 9: Table S6 in lists all primers used in this study. *Escherichia coli* biotin ligase (BirA) in pTG vector was used for the co-expression with GiPuf4 [80]. *G. intestinalis* transfection was performed as previously described [40]. Briefly, 1 × 10^7^ cells were electroporated with a Bio-Rad Gene Pulser using an exponential protocol (*U* = 350 V; *C* = 1,000 μF; *R* = 750 Ω). The transfected cells were grown in medium supplemented with antibiotics (57 μg/ml puromycin and 600 μg/ml G418).

### Cell fractionation

Trophozoites of *G. intestinalis* in logarithmic growth were harvested in ice-cold phosphate-buffered saline (PBS, pH 7.4) at 1000×*g* at 4 °C for 10 min, washed in SM buffer (20 mM MOPS, 250 mM sucrose, pH 7.4), and collected by centrifugation at 1000×*g*, for 10 min at 4 °C. The pellet of the cells was resuspended in SM buffer supplemented with protease inhibitors (Roche). The cells were lysed on ice by sonication with 1-s pulses and 40% amplitude for 2 min (Sonicator ultrasonic processor Q125, Qsonica). The lysate was subjected to centrifugation at 2680×*g*, for 20 min at 4 °C to sediment nuclei, cytoskeleton, and remaining unbroken cells. The supernatant was subjected to centrifugation at 180,000×*g*, for 30 min at 4 °C. The resulting supernatant corresponded to cytosolic fraction, and the high-speed pellet (HSP) contained organelles including the mitosomes and the endoplasmic reticulum.

### Crosslinking, protein isolation, and mass spectrometry (MS)

*G. intestinalis* cells were grown in TYI-S-33 medium enriched with 50 μM biotin for 24 h before harvesting. The cells were lysed by sonication, and the cell lysate (25 mg of total protein) was used for the protein isolation. The sample was diluted to final protein concentration of 3 mg/ml in PBS (pH 7.4) and supplemented with protease inhibitors (Roche). Crosslinker DSP (dithiobis [succinimidyl propionate]; Thermo Scientific) was added to the final concentration of 200 μM, and the sample was incubated 1 h on ice. The reaction was stopped by adding 50 mM final concentration of Tris (pH 8.0) for 15 min at room temperature. The sample was diluted 5 times with the boiling buffer (50 mM Tris, 1 mM EDTA, 1% SDS, pH 7.4), incubated at 80 °C for 10 min, and collected by centrifugation at 30,000×*g*, for 10 min at room temperature. Supernatant was diluted 1:10 in the binding buffer (50 mM Tris, 150 mM NaCl, 5 mM EDTA, 1% Triton X-100, pH 7.4). Meanwhile, 100 μl of streptavidin-coupled magnetic beads (Dynabeads MyOne Streptavidin C1, Invitrogen) was washed 3 times in the binding buffer using a magnetic stand (according to the instructions of the manufacturer). The beads were mixed with the supernatant, and the sample was incubated for 16 h at 4 °C with gentle rotation. The magnetic beads were washed 3 times in the binding buffer with 0.1% sodium deoxycholate (SDC) for 5 min, once in boiling buffer for 5 min, once in washing buffer (60 mM Tris, 2% SDC, 10% glycerol) for 5 min, and finally twice in 100 mM triethylammonium bicarbonate (TEAB) with 0.1% SDC for 5 min. One tenth of the beads was used to analyse the efficiency of the procedure. Specifically, the sample was mixed with the SDS-PAGE sample buffer containing 20 mM biotin and incubated at 95 °C for 5 min to elute the proteins from the beads. The samples were resolved on 12% SDS-PAGE and transferred to nitrocellulose membrane. For immunodetection, the following antibodies were used: 1° anti-BAP (1:1000) (Genscript) and 2° anti-rabbit polyclonal antibody coupled to HRP (1:2000) (Sigma). Remaining beads with bound proteins were submitted to mass spectrometry analysis.

### Tandem mass spectrometry (MS/MS) analyses

The beads were incubated at 5 mM tris (2-carboxyethyl) phosphine (TCEP) at 60 °C for 1 h to reduce the disulfide bridges and blocked with 10 mM final concentration of methyl methanethiosulfonate (MMTS) for 10 min at 37 °C. Samples were cleaved on the beads with 1 μg of trypsin at 37 °C for 16 h. After digestion, samples were centrifuged, and supernatants were collected and acidified with trifluoroacetic acid (TFA at a final concentration of 1%). SDC was removed by extraction with ethylacetate [81]. Peptides were desalted on Michrom C18 column. Nano Reversed phase column (EASY-Spray column, 50 cm × 75 μm ID, PepMap C18, 2 μm particles, 100 Å pore size) was used for LC/MS analysis. Mobile phase buffer A was composed of water and 0.1% formic acid. Mobile phase B was composed of 0.1% formic acid in acetonitrile. Samples were loaded onto the trap column (Acclaim PepMap300, C18, 5 μm, 300 Å Wide Pore, 300 μm × 5 mm, 5 Cartridges) for 4 min at 15 μl/min. Loading buffer was composed of water, 2% acetonitrile, and 0.1% TFA. Peptides were eluted with mobile phase B gradient from 4 to 35% B in 1 h. Eluting peptide cations were converted to gas-phase ions by electrospray ionization and analysed on a Thermo Orbitrap Fusion (Q-OT-qIT, Thermo). Survey scans of peptide precursors from 400 to 1600 m/z were performed at 120 K resolution (at 200 m/z) with a 5 × 10^5^ ion count target. Tandem MS was performed by isolation at 1.5 Th with the quadrupole, HCD fragmentation with normalized collision energy of 30, and rapid scan MS analysis in the ion trap. The MS 2 ion count target was set to 104, and the max injection time was 35 ms. Only those precursors with charge state 2–6 were sampled for MS 2. The dynamic exclusion duration was set to 45 s with a 10-ppm tolerance around the selected precursor and its isotopes. Monoisotopic precursor selection was turned on. The instrument was run in top speed mode with 2-s cycles [82]. All data were analysed and quantified with the MaxQuant software (version 1.5.3.8) [83]. The false discovery rate (FDR) was set to 1% for both proteins and peptides, and we specified a minimum length of seven amino acids. The Andromeda search engine was used for the MS/MS spectra search against the *G. intestinalis* database (downloaded from UniProtKB in September 2017, containing 12,665 entries). Enzyme specificity was set as C-terminal to Arg and Lys, also allowing cleavage at proline bonds and a maximum of two missed cleavages. Dithiomethylation of cysteine was selected as fixed modification and N-terminal protein acetylation and methionine oxidation as variable modifications. The ‘match between runs’ feature of MaxQuant was used to transfer identifications to other LC-MS/MS runs based on their masses and retention time (maximum deviation 0.7 min), and this was also used in quantification experiments. Quantifications were performed with the label-free algorithms described recently [83]. Data analysis was performed using Perseus 1.5.2.4 software [84]. The mass spectrometry proteomics data have been deposited in the ProteomeXchange Consortium via the PRIDE [85] partner repository with the dataset identifier PXD019608.

### Immunofluorescence microscopy

*G. intestinalis* trophozoites were fixed in 1% paraformaldehyde for 30 min at 37 °C and collected by centrifugation at 1000×*g* for 5 min at room temperature. The cells were washed in PEM buffer (100 mM PIPES pH 6.9, 1 mM EGTA, and 0.1 mM MgSO_4_) and placed on cover slips. The cells were permeabilized by 0.2% Triton X-100 (Sigma-Aldrich) for 20 min, washed three times with 1× PEM buffer, and incubated with primary antibodies in PEMBALG (100 mM PIPES pH 6.9, 1 mM EGTA, 0.1 mM MgSO_4_, 1% BSA, 0.1% NaN_3_, 100 mM lysine, and 0.5% cold-water fish skin gelatin) for 1 h. Cells were probed with the following primary antibodies: rat anti-HA monoclonal IgG antibody (1:1000 dilution), mouse anti-BAP monoclonal antibody (1:1000 dilution), and rabbit anti-Sec20 polyclonal [86] (1:1000 dilution). The cover slips were washed three times with 1 ml of 1× PEM and were incubated with secondary antibodies Alexa Fluor 488-conjugated goat anti-rat IgG and Alexa 594-conjugated donkey anti-rabbit IgG or Alexa594-conjugated goat anti-mouse IgG for 1 h. After 3 × 5 min washes in PEM buffer, slides were mounted with Vectashield containing DAPI (4′, 6-diamidino-2-phenylindole; Vector Laboratories) or Hoechst solution (33258). Stimulated emission depletion (STED) microscopy was performed on a commercial Abberior STED 775 QUAD scanning microscope (Abberior Instruments GmbH, Germany) equipped with Ti-E Nikon body, QUAD beam scanner, Easy3D STED Optics Module, and Nikon CFI Plan Apo Lambda objective (× 60 Oil, NA 1.40). Samples were illuminated by pulsed 561-nm and 640-nm lasers and depleted by a pulsed 775-nm STED laser of 2D donut shape (all lasers: 40 MHz repetition rate). Fluorescence signal was detected with single photon counting modules (Excelitas Technologies). Line-interleaved acquisition enabled separated detection of individual channels in spectral range from 605 to 625 nm and from 650 to 720 nm. The confocal pinhole was set to 1 AU.

## Supplementary information

**Additional file 1: Table S1.** The clustering analysis of PufSF proteins.

**Additional file 2: Table S2.** Classification of PufSF proteins from UniProt referenced proteomes.

**Additional file 3: Figure S1.** Phylogenetic analysis NOP9, PUM3 and PUF proteins. Full phylogenies of maximum-likelihood analysis of PUM3, Nop9 and Puf proteins with SH-like approximate likelihood ratio test, ultrafast bootstrap supports, and with both non-parametric Felsenstein’s Bootstrap Proportion (FBP) supports (i.e.*,* PMSF) and Transfer Bootstrap Expectation (TBE) supports as indicated. See Additional file 7 for alignment properties and model parameters.

**Additional file 4: Figure S2.** Proteins sequence alignment of Nop9 orthologues. **Figure S3.** Protein sequence alignment of PUM3 orthologues.

**Additional file 5: Figure S4.** Volcano plot of proteomics analysis of GiPuf4 pull down.

**Additional file 6: Table S3.** Proteomics analysis of GiPuf4 pull down.

**Additional file 7: Table S4.** Alignment properties and model parameters used for the phylogenetic analyses.

**Additional file 8: Table S5.** Prediction of *Giardia* Pufs target mRNA.

**Additional file 9: Table S6.** Primers used in the study.

## Data Availability

All data generated or analysed during this study are included in this published article and its Additional files or deposited online. The mass spectrometry proteomics data have been deposited in the ProteomeXchange Consortium via the PRIDE [85] partner repository with the dataset identifier PXD019608. Phylogenetic datasets are available on the figshare repository (DOI:10.6084/m9.figshare.12097692) [87].
