# Supplementary material for: The evolution of the Puf superfamily of proteins across the tree of eukaryotes
Source: BMC Biol. 2020 Jun 30;18:77. doi: 10.1186/s12915-020-00814-3 (PMC7325665; doi:10.1186/s12915-020-00814-3)
Supplement: Supplementary file 3 — Additional file 3: Figure S1. Phylogenetic analysis NOP9, PUM3 and PUF proteins. Full phylogenies of maximum-likelihood analysis of PUM3, Nop9 and Puf proteins with SH-like approximate likelihood ratio test, ultrafast bootstrap supports, and with both non-parametric Felsenstein’s Bootstrap Proportion (FBP) supports (i.e., PMSF) and Transfer Bootstrap Expectation (TBE) supports as indicated. See Additional file 7 for alignment properties and model parameters. [file 12915_2020_814_MOESM3_ESM.pdf]

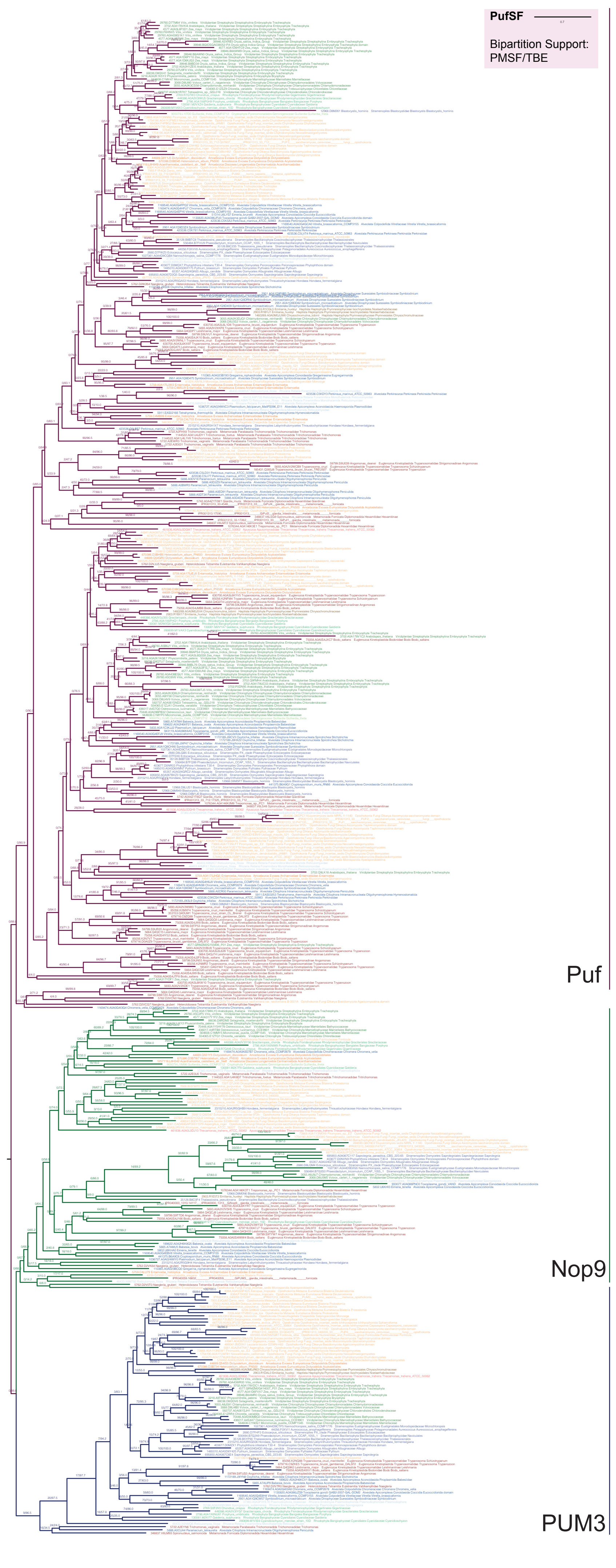
PufSF
Bipartition Support:
PMSF/TBE
Puf
Nop9
PUM3

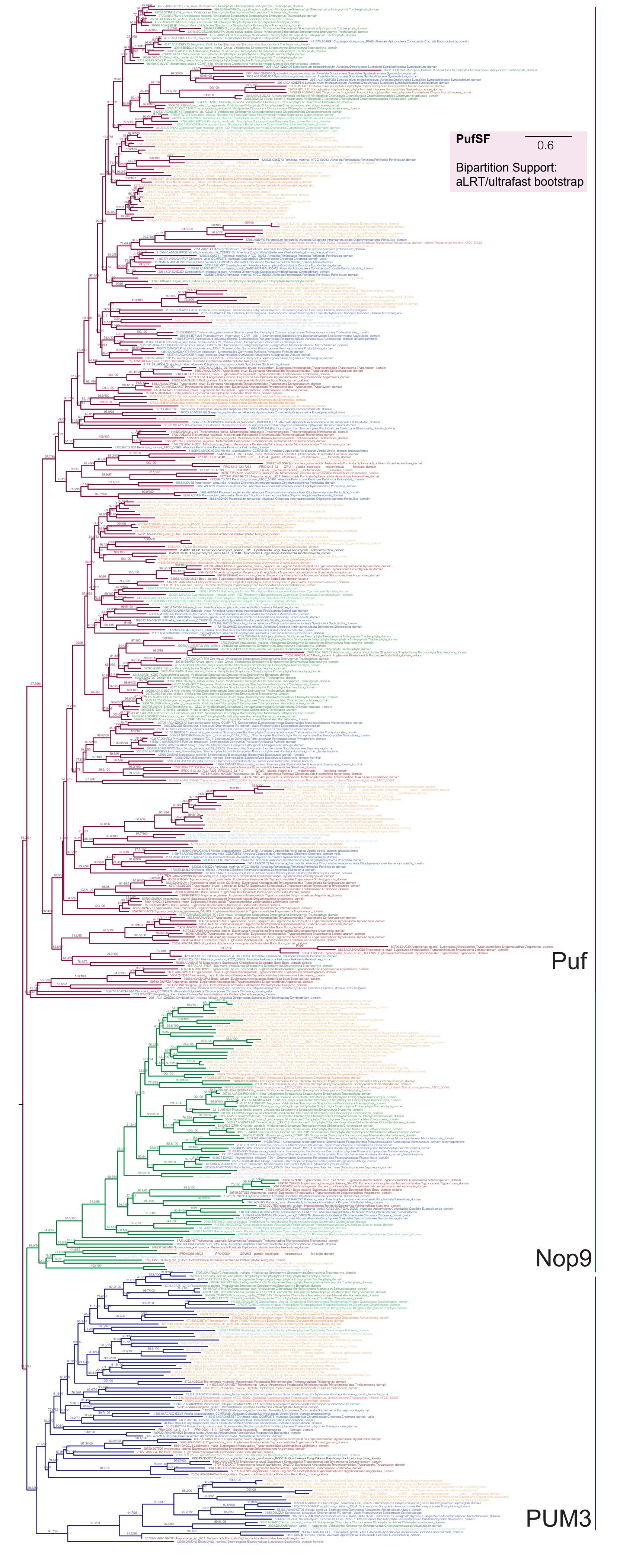
PufSF
0.6
Bipartition Support:
aLRT/ultrafast bootstrap
Puf
Nop9
PUM3

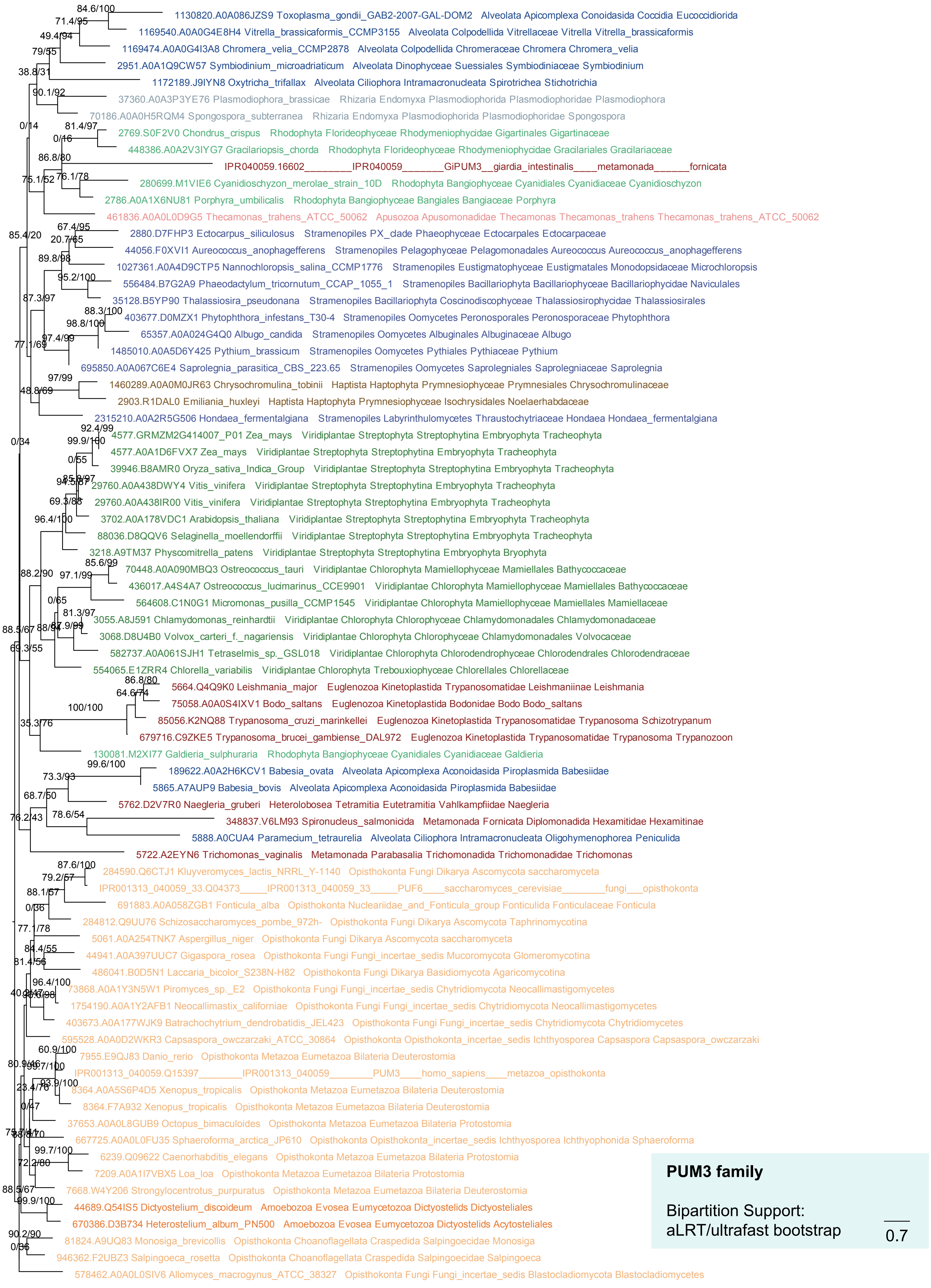
1130820.A0A086JZS9 Toxoplasma_gondii_GAB2-2007-GAL-DOM2 Alveolata Apicomplexa Conoidasida Coccidia Eucoccidiorida
1169540.A0A0G4E8H4 Vitrella_brassicaformis_CCMP3155 Alveolata Colpodellida Vitrellaceae Vitrella Vitrella_brassicaformis
1169474.A0A0G4I3A8 Chromera_velia_CCMP2878 Alveolata Colpodellida Chromeraceae Chromera Chromera_velia
2951.A0A1Q9CW57 Symbiodinium_microadriaticum Alveolata Dinophyceae Suessiales Symbiodiniaceae Symbiodinium
1172189.J9IYN8 Oxytricha_trifallax Alveolata Ciliophora Intramacronucleata Spirotrichea Stichotrichia
37360.A0A3P3YE76 Plasmodiophora_brassicae Rhizaria Endomyxa Plasmodiophorida Plasmodiophoridae Plasmodiophora
70186.A0A0H5RQM4 Spongospora_subterranea Rhizaria Endomyxa Plasmodiophorida Plasmodiophoridae Spongospora
2769.S0F2V0 Chondrus_crispus Rhodophyta Florideophyceae Rhodymeniophycidae Gigartinales Gigartinaceae
448386.A0A2V3IYG7 Gracilariopsis_chorda Rhodophyta Florideophyceae Rhodymeniophycidae Gracilariales Gracilariaceae
IPR040059.16602_________IPR040059________GiPUM3__giardia_intestinalis____metamonada_______fornicata
280699.M1VIE6 Cyanidioschyzon_merolae_strain_10D Rhodophyta Bangiophyceae Cyanidiales Cyanidiaceae Cyanidioschyzon
2786.A0A1X6NU81 Porphyra_umbilicalis Rhodophyta Bangiophyceae Bangiales Bangiaceae Porphyra
461836.A0A0L0D9G5 Thecamonas_trahens_ATCC_50062 Apusozoa Apusomonadidae Thecamonas Thecamonas_trahens Thecamonas_trahens_ATCC_50062
2880.D7FHP3 Ectocarpus_siliculosus Stramenopiles PX_clade Phaeophyceae Ectocarpales Ectocarpaceae
44056.F0XVI1 Aureococcus_anophagefferens Stramenopiles Pelagophyceae Pelagomonadales Aureococcus Aureococcus_anophagefferens
1027361.A0A4D9CTP5 Nannochloropsis_salina_CCMP1776 Stramenopiles Eustigmatophyceae Eustigmatales Monodopsidaceae Microchloropsis
556484.B7G2A9 Phaeodactylum_tricornutum_CCAP_1055_1 Stramenopiles Bacillariophyta Bacillariophyceae Bacillariophycidae Naviculales
35128.B5YP90 Thalassiosira_pseudonana Stramenopiles Bacillariophyta Coscinodiscophyceae Thalassiosirophycidae Thalassiosirales
403677.D0MZX1 Phytophthora_infestans_T30-4 Stramenopiles Oomycetes Peronosporales Peronosporaceae Phytophthora
65357.A0A024G4Q0 Albugo_candida Stramenopiles Oomycetes Albuginales Albuginaceae Albugo
1485010.A0A5D6Y425 Pythium_brassicum Stramenopiles Oomycetes Pythiales Pythiaceae Pythium
695850.A0A067C6E4 Saprolegnia_parasitica_CBS_223.65 Stramenopiles Oomycetes Saprolegniales Saprolegniaceae Saprolegnia
1460289.A0A0M0JR63 Chrysochromulina_tobinii Haptista Haptophyta Prymnesiophyceae Prymnesiales Chrysochromulinaceae
2903.R1DAL0 Emiliania_huxleyi Haptista Haptophyta Prymnesiophyceae Isochrysidales Noelaerhabdaceae
2315210.A0A2R5G506 Hondaea_fermentalgiana Stramenopiles Labyrinthulomycetes Thraustochytriaceae Hondaea Hondaea_fermentalgiana
4577.GRMZM2G414007_P01 Zea_mays Viridiplantae Streptophyta Streptophytina Embryophyta Tracheophyta
4577.A0A1D6FVX7 Zea_mays Viridiplantae Streptophyta Streptophytina Embryophyta Tracheophyta
39946.B8AMR0 Oryza_sativa_Indica_Group Viridiplantae Streptophyta Streptophytina Embryophyta Tracheophyta
29760.A0A438DWY4 Vitis_vinifera Viridiplantae Streptophyta Streptophytina Embryophyta Tracheophyta
29760.A0A438IR00 Vitis_vinifera Viridiplantae Streptophyta Streptophytina Embryophyta Tracheophyta
3702.A0A178VDC1 Arabidopsis_thaliana Viridiplantae Streptophyta Streptophytina Embryophyta Tracheophyta
88036.D8QQV6 Selaginella_moellendorffii Viridiplantae Streptophyta Streptophytina Embryophyta Tracheophyta
3218.A9TM37 Physcomitrella_patens Viridiplantae Streptophyta Streptophytina Embryophyta Bryophyta
70448.A0A090MBQ3 Ostreococcus_tauri Viridiplantae Chlorophyta Mamiellophyceae Mamiellales Bathycoccaceae
436017.A4S4A7 Ostreococcus_lucimarinus_CCE9901 Viridiplantae Chlorophyta Mamiellophyceae Mamiellales Bathycoccaceae
564608.C1N0G1 Micromonas_pusilla_CCMP1545 Viridiplantae Chlorophyta Mamiellophyceae Mamiellales Mamiellaceae
3055.A8J591 Chlamydomonas_reinhardtii Viridiplantae Chlorophyta Chlorophyceae Chlamydomonadales Chlamydomonadaceae
3068.D8U4B0 Volvox_carteri_f._nagariensis Viridiplantae Chlorophyta Chlorophyceae Chlamydomonadales Volvocaceae
582737.A0A061SJH1 Tetraselmis_sp._GSL018 Viridiplantae Chlorophyta Chlorodendrophyceae Chlorodendrales Chlorodendraceae
554065.E1ZRR4 Chlorella_variabilis Viridiplantae Chlorophyta Trebouxiophyceae Chlorellales Chlorellaceae
5664.Q4Q9K0 Leishmania_major Euglenozoa Kinetoplastida Trypanosomatidae Leishmaniinae Leishmania
75058.A0A0S4IXV1 Bodo_saltans Euglenozoa Kinetoplastida Bodonidae Bodo Bodo_saltans
85056.K2NQ88 Trypanosoma_cruzi_marinkellei Euglenozoa Kinetoplastida Trypanosomatidae Trypanosoma Schizotrypanum
679716.C9ZKE5 Trypanosoma_brucei_gambiense_DAL972 Euglenozoa Kinetoplastida Trypanosomatidae Trypanosoma Trypanozoon
130081.M2XI77 Galdieria_sulphuraria Rhodophyta Bangiophyceae Cyanidiales Cyanidiaceae Galdieria
189622.A0A2H6KCV1 Babesia_ovata Alveolata Apicomplexa Aconoidasida Piroplasmida Babesiidae
5865.A7AUP9 Babesia_bovis Alveolata Apicomplexa Aconoidasida Piroplasmida Babesiidae
5762.D2V7R0 Naegleria_gruberi Heterolobosea Tetramitia Eutetramitia Vahlkampfiidae Naegleria
348837.V6LM93 Spironucleus_salmonicida Metamonada Fornicata Diplomonadida Hexamitidae Hexamitinae
5888.A0CUA4 Paramecium_tetraurelia Alveolata Ciliophora Intramacronucleata Oligohymenophorea Peniculida
5722.A2EYN6 Trichomonas_vaginalis Metamonada Parabasalia Trichomonadida Trichomonadidae Trichomonas
284590.Q6CTJ1 Kluyveromyces_lactis_NRRL_Y-1140 Opisthokonta Fungi Dikarya Ascomycota saccharomyceta
IPR001313_040059_33.Q04373______IPR001313_040059_33______PUF6_____saccharomyces_cerevisiae_________fungi____opisthokonta
691883.A0A058ZGB1 Fonticula_alba Opisthokonta Nucleariidae_and_Fonticula_group Fonticulida Fonticulaceae Fonticula
284812.Q9UU76 Schizosaccharomyces_pombe_972h- Opisthokonta Fungi Dikarya Ascomycota Taphrinomycotina
5061.A0A254TNK7 Aspergillus_niger Opisthokonta Fungi Dikarya Ascomycota saccharomyceta
44941.A0A397UUC7 Gigaspora_rosea Opisthokonta Fungi Fungi_incertae_sedis Mucoromycota Glomeromycotina
486041.B0D5N1 Laccaria_bicolor_S238N-H82 Opisthokonta Fungi Dikarya Basidiomycota Agaricomycotina
73868.A0A1Y3N5W1 Piromyces_sp._E2 Opisthokonta Fungi Fungi_incertae_sedis Chytridiomycota Neocallimastigomycetes
1754190.A0A1Y2AFB1 Neocallimastix_californiae Opisthokonta Fungi Fungi_incertae_sedis Chytridiomycota Neocallimastigomycetes
403673.A0A177WJK9 Batrachochytrium_dendrobatidis_JEL423 Opisthokonta Fungi Fungi_incertae_sedis Chytridiomycota Chytridiomycetes
595528.A0A0D2WKR3 Capsaspora_owczarzaki_ATCC_30864 Opisthokonta Opisthokonta_incertae_sedis Ichthyosporea Capsaspora Capsaspora_owczarzaki
7955.E9QJ83 Danio_rerio Opisthokonta Metazoa Eumetazoa Bilateria Deuterostomia
IPR001313_040059.Q15397_________IPR001313_040059_________PUM3_____homo_sapiens_____metazoa_opisthokonta
8364.A0A5S6P4D5 Xenopus_tropicalis Opisthokonta Metazoa Eumetazoa Bilateria Deuterostomia
8364.F7A932 Xenopus_tropicalis Opisthokonta Metazoa Eumetazoa Bilateria Deuterostomia
37653.A0A0L8GUB9 Octopus_bimaculoides Opisthokonta Metazoa Eumetazoa Bilateria Protostomia
667725.A0A0L0FU35 Sphaeroforma_arctica_JP610 Opisthokonta Opisthokonta_incertae_sedis Ichthyosporea Ichthyophonida Sphaeroforma
6239.Q09622 Caenorhabditis_elegans Opisthokonta Metazoa Eumetazoa Bilateria Protostomia
7209.A0A1I7VBX5 Loa_loa Opisthokonta Metazoa Eumetazoa Bilateria Protostomia
7668.W4Y206 Strongylocentrotus_purpuratus Opisthokonta Metazoa Eumetazoa Bilateria Deuterostomia
44689.Q54IS5 Dictyostelium_discoideum Amoebozoa Evosea Eumycetozoa Dictyostelids Dictyosteliales
670386.D3B734 Heterostelium_album_PN500 Amoebozoa Evosea Eumycetozoa Dictyostelids Acytosteliales
81824.A9UQ83 Monosiga_brevicollis Opisthokonta Choanoflagellata Craspedida Salpingoecidae Monosiga
946362.F2UBZ3 Salpingoeca_rosetta Opisthokonta Choanoflagellata Craspedida Salpingoecidae Salpingoeca
578462.A0A0L0SIV6 Allomyces_macrogynus_ATCC_38327 Opisthokonta Fungi Fungi_incertae_sedis Blastocladiomycota Blastocladiomycetes
PUM3 family
Bipartition Support:
aLRT/ultrafast bootstrap
0.7

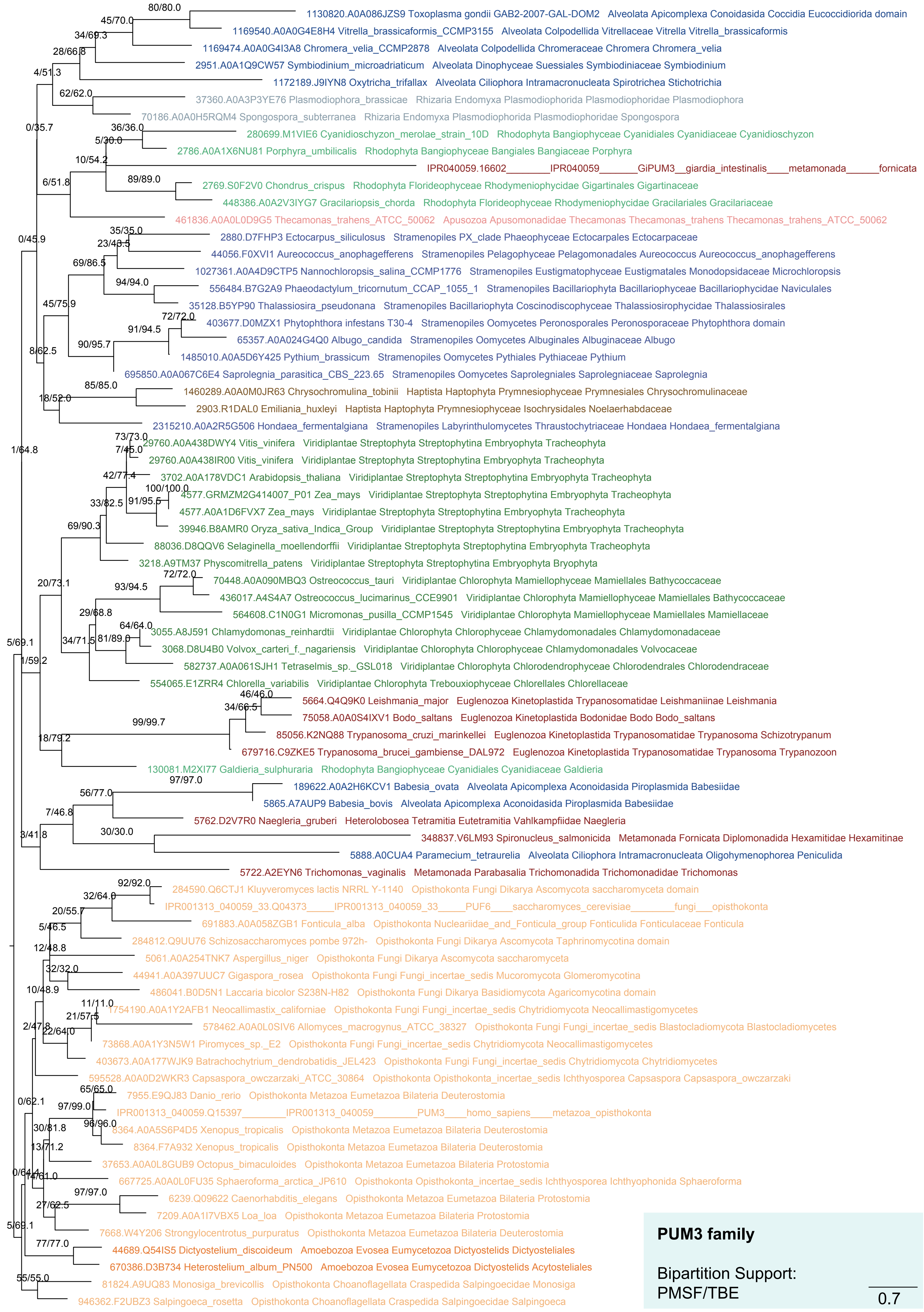
1130820.A0A086JZS9 Toxoplasma gondii GAB2-2007-GAL-DOM2 Alveolata Apicomplexa Conoidasida Coccidia Eucoccidiorida domain
1169540.A0A0G4E8H4 Vitrella_brassicaformis_CCMP3155 Alveolata Colpodellida Vitrellaceae Vitrella Vitrella_brassicaformis
1169474.A0A0G4I3A8 Chromera_velia_CCMP2878 Alveolata Colpodellida Chromeraceae Chromera Chromera_velia
2951.A0A1Q9CW57 Symbiodinium_microadriaticum Alveolata Dinophyceae Suessiales Symbiodiniaceae Symbiodinium
1172189.J9IYN8 Oxytricha_trifallax Alveolata Ciliophora Intramacronucleata Spirotrichea Stichotrichia
37360.A0A3P3YE76 Plasmodiophora_brassicae Rhizaria Endomyxa Plasmodiophorida Plasmodiophoridae Plasmodiophora
70186.A0A0H5RQM4 Spongospora_subterranea Rhizaria Endomyxa Plasmodiophorida Plasmodiophoridae Spongospora
280699.M1VIE6 Cyanidioschyzon_merolae_strain_10D Rhodophyta Bangiophyceae Cyanidiales Cyanidiaceae Cyanidioschyzon
2786.A0A1X6NU81 Porphyra_umbilicalis Rhodophyta Bangiophyceae Bangiales Bangiaceae Porphyra
IPR040059.16602________IPR040059_______GiPUM3__giardia_intestinalis____metamonada______fornicata
2769.S0F2V0 Chondrus_crispus Rhodophyta Florideophyceae Rhodymeniophycidae Gigartinales Gigartinaceae
448386.A0A2V3IYG7 Gracilariopsis_chorda Rhodophyta Florideophyceae Rhodymeniophycidae Gracilariales Gracilariaceae
461836.A0A0L0D9G5 Thecamonas_trahens_ATCC_50062 Apusozoa Apusomonadidae Thecamonas Thecamonas_trahens Thecamonas_trahens_ATCC_50062
2880.D7FHP3 Ectocarpus_siliculosus Stramenopiles PX_clade Phaeophyceae Ectocarpales Ectocarpaceae
44056.F0XVI1 Aureococcus_anophagefferens Stramenopiles Pelagophyceae Pelagomonadales Aureococcus Aureococcus_anophagefferens
1027361.A0A4D9CTP5 Nannochloropsis_salina_CCMP1776 Stramenopiles Eustigmatophyceae Eustigmatales Monodopsidaceae Microchloropsis
556484.B7G2A9 Phaeodactylum_tricornutum_CCAP_1055_1 Stramenopiles Bacillariophyta Bacillariophyceae Bacillariophycidae Naviculales
35128.B5YP90 Thalassiosira_pseudonana Stramenopiles Bacillariophyta Coscinodiscophyceae Thalassiosirophycidae Thalassiosirales
403677.D0MZX1 Phytophthora infestans T30-4 Stramenopiles Oomycetes Peronosporales Peronosporaceae Phytophthora domain
65357.A0A024G4Q0 Albugo_candida Stramenopiles Oomycetes Albuginales Albuginaceae Albugo
1485010.A0A5D6Y425 Pythium_brassicum Stramenopiles Oomycetes Pythiales Pythiaceae Pythium
695850.A0A067C6E4 Saprolegnia_parasitica_CBS_223.65 Stramenopiles Oomycetes Saprolegniales Saprolegniaceae Saprolegnia
1460289.A0A0M0JR63 Chrysochromulina_tobinii Haptista Haptophyta Prymnesiophyceae Prymnesiales Chrysochromulinaceae
2903.R1DAL0 Emiliania_huxleyi Haptista Haptophyta Prymnesiophyceae Isochrysidales Noelaerhabdaceae
2315210.A0A2R5G506 Hondaea_fermentalgiana Stramenopiles Labyrinthulomycetes Thraustochytriaceae Hondaea Hondaea_fermentalgiana
29760.A0A438DWY4 Vitis_vinifera Viridiplantae Streptophyta Streptophytina Embryophyta Tracheophyta
29760.A0A438IR00 Vitis_vinifera Viridiplantae Streptophyta Streptophytina Embryophyta Tracheophyta
3702.A0A178VDC1 Arabidopsis_thaliana Viridiplantae Streptophyta Streptophytina Embryophyta Tracheophyta
4577.GRMZM2G414007_P01 Zea_mays Viridiplantae Streptophyta Streptophytina Embryophyta Tracheophyta
4577.A0A1D6FVX7 Zea_mays Viridiplantae Streptophyta Streptophytina Embryophyta Tracheophyta
39946.B8AMR0 Oryza_sativa_Indica_Group Viridiplantae Streptophyta Streptophytina Embryophyta Tracheophyta
88036.D8QQV6 Selaginella_moellendorffii Viridiplantae Streptophyta Streptophytina Embryophyta Tracheophyta
3218.A9TM37 Physcomitrella_patens Viridiplantae Streptophyta Streptophytina Embryophyta Bryophyta
70448.A0A090MBQ3 Ostreococcus_tauri Viridiplantae Chlorophyta Mamiellophyceae Mamiellales Bathycoccaceae
436017.A4S4A7 Ostreococcus_lucimarinus_CCE9901 Viridiplantae Chlorophyta Mamiellophyceae Mamiellales Bathycoccaceae
564608.C1N0G1 Micromonas_pusilla_CCMP1545 Viridiplantae Chlorophyta Mamiellophyceae Mamiellales Mamiellaceae
3055.A8J591 Chlamydomonas_reinhardtii Viridiplantae Chlorophyta Chlorophyceae Chlamydomonadales Chlamydomonadaceae
3068.D8U4B0 Volvox_carteri_f._nagariensis Viridiplantae Chlorophyta Chlorophyceae Chlamydomonadales Volvocaceae
582737.A0A061SJH1 Tetraselmis_sp._GSL018 Viridiplantae Chlorophyta Chlorodendrophyceae Chlorodendrales Chlorodendraceae
554065.E1ZRR4 Chlorella_variabilis Viridiplantae Chlorophyta Trebouxiophyceae Chlorellales Chlorellaceae
5664.Q4Q9K0 Leishmania_major Euglenozoa Kinetoplastida Trypanosomatidae Leishmaniinae Leishmania
75058.A0A0S4IXV1 Bodo_saltans Euglenozoa Kinetoplastida Bodonidae Bodo Bodo_saltans
85056.K2NQ88 Trypanosoma_cruzi_marinkellei Euglenozoa Kinetoplastida Trypanosomatidae Trypanosoma Schizotrypanum
679716.C9ZKE5 Trypanosoma_brucei_gambiense_DAL972 Euglenozoa Kinetoplastida Trypanosomatidae Trypanosoma Trypanozoon
130081.M2XI77 Galdieria_sulphuraria Rhodophyta Bangiophyceae Cyanidiales Cyanidiaceae Galdieria
189622.A0A2H6KCV1 Babesia_ovata Alveolata Apicomplexa Aconoidasida Piroplasmida Babesiidae
5865.A7AUP9 Babesia_bovis Alveolata Apicomplexa Aconoidasida Piroplasmida Babesiidae
5762.D2V7R0 Naegleria_gruberi Heterolobosea Tetramitia Eutetramitia Vahlkampfiidae Naegleria
348837.V6LM93 Spironucleus_salmonicida Metamonada Fornicata Diplomonadida Hexamitidae Hexamitinae
5888.A0CUA4 Paramecium_tetraurelia Alveolata Ciliophora Intramacronucleata Oligohymenophorea Peniculida
5722.A2EYN6 Trichomonas_vaginalis Metamonada Parabasalia Trichomonadida Trichomonadidae Trichomonas
284590.Q6CTJ1 Kluyveromyces lactis NRRL Y-1140 Opisthokonta Fungi Dikarya Ascomycota saccharomyceta domain
IPR001313_040059_33.Q04373_____IPR001313_040059_33_____PUF6____saccharomyces_cerevisiae________fungi___opisthokonta
691883.A0A058ZGB1 Fonticula_alba Opisthokonta Nucleariidae_and_Fonticula_group Fonticulida Fonticulaceae Fonticula
284812.Q9UU76 Schizosaccharomyces pombe 972h- Opisthokonta Fungi Dikarya Ascomycota Taphrinomycotina domain
5061.A0A254TNK7 Aspergillus_niger Opisthokonta Fungi Dikarya Ascomycota saccharomyceta
44941.A0A397UUC7 Gigaspora_rosea Opisthokonta Fungi Fungi_incertae_sedis Mucoromycota Glomeromycotina
486041.B0D5N1 Laccaria bicolor S238N-H82 Opisthokonta Fungi Dikarya Basidiomycota Agaricomycotina domain
1754190.A0A1Y2AFB1 Neocallimastix_californiae Opisthokonta Fungi Fungi_incertae_sedis Chytridiomycota Neocallimastigomycetes
578462.A0A0L0SIV6 Allomyces_macrogynus_ATCC_38327 Opisthokonta Fungi Fungi_incertae_sedis Blastocladiomycota Blastocladiomycetes
73868.A0A1Y3N5W1 Piromyces_sp._E2 Opisthokonta Fungi Fungi_incertae_sedis Chytridiomycota Neocallimastigomycetes
403673.A0A177WJK9 Batrachochytrium_dendrobatidis_JEL423 Opisthokonta Fungi Fungi_incertae_sedis Chytridiomycota Chytridiomycetes
595528.A0A0D2WKR3 Capsaspora_owczarzaki_ATCC_30864 Opisthokonta Opisthokonta_incertae_sedis Ichthyosporea Capsaspora Capsaspora_owczarzaki
7955.E9QJ83 Danio_rerio Opisthokonta Metazoa Eumetazoa Bilateria Deuterostomia
IPR001313_040059.Q15397________IPR001313_040059________PUM3____homo_sapiens____metazoa_opisthokonta
8364.A0A5S6P4D5 Xenopus_tropicalis Opisthokonta Metazoa Eumetazoa Bilateria Deuterostomia
8364.F7A932 Xenopus_tropicalis Opisthokonta Metazoa Eumetazoa Bilateria Deuterostomia
37653.A0A0L8GUB9 Octopus_bimaculoides Opisthokonta Metazoa Eumetazoa Bilateria Protostomia
667725.A0A0L0FU35 Sphaeroforma_arctica_JP610 Opisthokonta Opisthokonta_incertae_sedis Ichthyosporea Ichthyophonida Sphaeroforma
6239.Q09622 Caenorhabditis_elegans Opisthokonta Metazoa Eumetazoa Bilateria Protostomia
7209.A0A1I7VBX5 Loa_loa Opisthokonta Metazoa Eumetazoa Bilateria Protostomia
7668.W4Y206 Strongylocentrotus_purpuratus Opisthokonta Metazoa Eumetazoa Bilateria Deuterostomia
44689.Q54IS5 Dictyostelium_discoideum Amoebozoa Evosea Eumycetozoa Dictyostelids Dictyosteliales
670386.D3B734 Heterostelium_album_PN500 Amoebozoa Evosea Eumycetozoa Dictyostelids Acytosteliales
81824.A9UQ83 Monosiga_brevicollis Opisthokonta Choanoflagellata Craspedida Salpingoecidae Monosiga
946362.F2UBZ3 Salpingoeca_rosetta Opisthokonta Choanoflagellata Craspedida Salpingoecidae Salpingoeca
PUM3 family
Bipartition Support:
PMSF/TBE
0.7

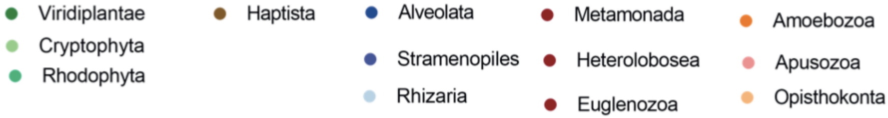
Viridiplantae
Cryptophyta
Rhodophyta
Haptista
Alveolata
Stramenopiles
Rhizaria
Metamonada
Heterolobosea
Euglenozoa
Amoebozoa
Apusozoa
Opisthokonta

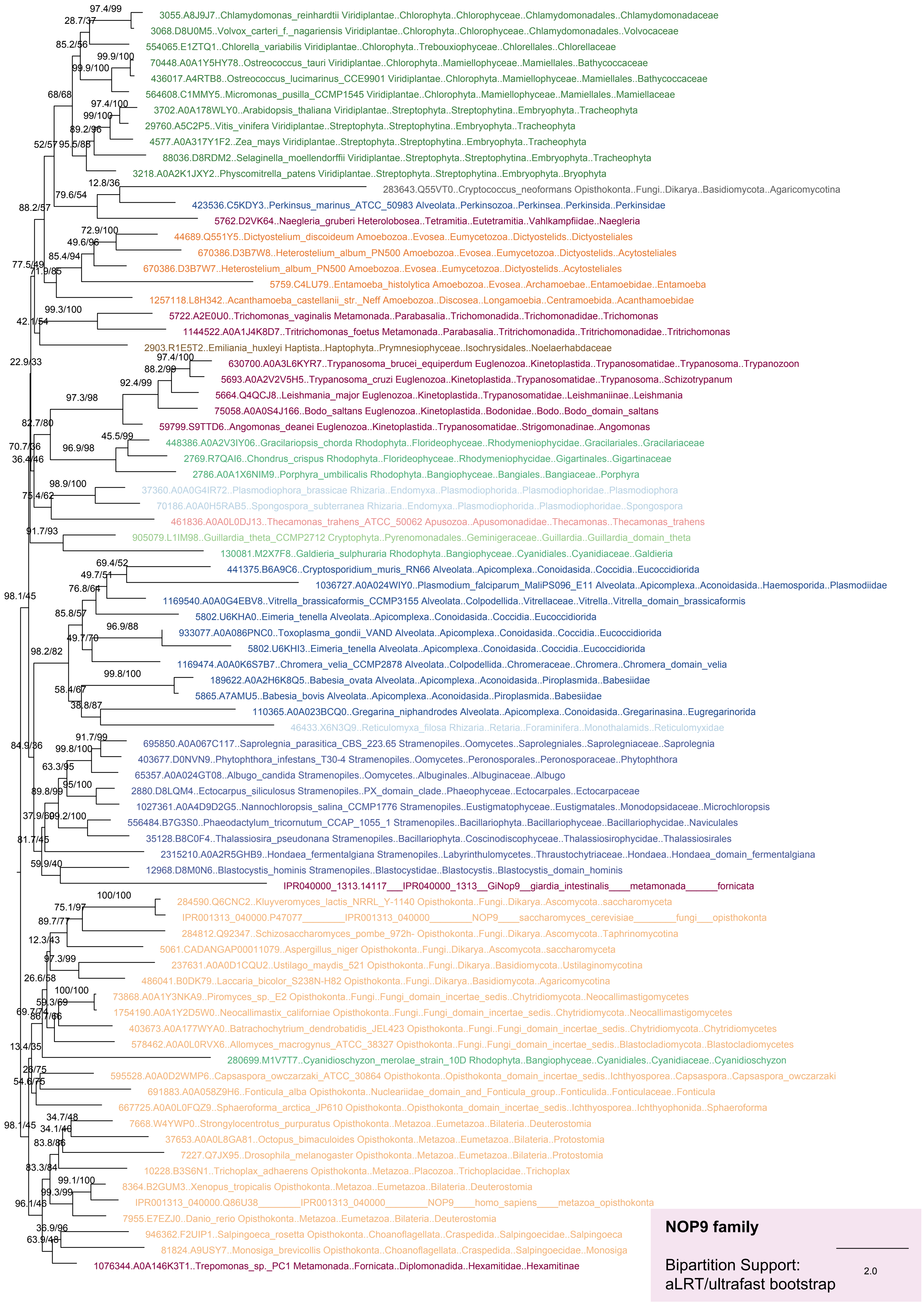
97.4/99
28.7/37
3055.A8J9J7..Chlamydomonas_reinhardtii Viridiplantae..Chlorophyta..Chlorophyceae..Chlamydomonadales..Chlamydomonadaceae
3068.D8U0M5..Volvox_carteri_f._nagariensis Viridiplantae..Chlorophyta..Chlorophyceae..Chlamydomonadales..Volvocaceae
85.2/56
554065.E1ZTQ1..Chlorella_variabilis Viridiplantae..Chlorophyta..Trebouxiophyceae..Chlorellales..Chlorellaceae
99.9/100
70448.A0A1Y5HY78..Ostreococcus_tauri Viridiplantae..Chlorophyta..Mamiellophyceae..Mamiellales..Bathycoccaceae
99.9/100
436017.A4RTB8..Ostreococcus_lucimarinus_CCE9901 Viridiplantae..Chlorophyta..Mamiellophyceae..Mamiellales..Bathycoccaceae
68/68
564608.C1MMY5..Micromonas_pusilla_CCMP1545 Viridiplantae..Chlorophyta..Mamiellophyceae..Mamiellales..Mamiellaceae
97.4/100
3702.A0A178WLY0..Arabidopsis_thaliana Viridiplantae..Streptophyta..Streptophytina..Embryophyta..Tracheophyta
99/100
29760.A5C2P5..Vitis_vinifera Viridiplantae..Streptophyta..Streptophytina..Embryophyta..Tracheophyta
89.2/96
4577.A0A317Y1F2..Zea_mays Viridiplantae..Streptophyta..Streptophytina..Embryophyta..Tracheophyta
52/57
95.5/88
88036.D8RDM2..Selaginella_moellendorffii Viridiplantae..Streptophyta..Streptophytina..Embryophyta..Tracheophyta
3218.A0A2K1JXY2..Physcomitrella_patens Viridiplantae..Streptophyta..Streptophytina..Embryophyta..Bryophyta
12.8/36
283643.Q55VT0..Cryptococcus_neoformans Opisthokonta..Fungi..Dikarya..Basidiomycota..Agaricomycotina
79.6/54
423536.C5KDY3..Perkinsus_marinus_ATCC_50983 Alveolata..Perkinsozoa..Perkinsea..Perkinsida..Perkinsidae
88.2/57
5762.D2VK64..Naegleria_gruberi Heterolobosea..Tetramitia..Eutetramitia..Vahlkampfiidae..Naegleria
72.9/100
44689.Q551Y5..Dictyostelium_discoideum Amoebozoa..Evosea..Eumycetozoa..Dictyostelids..Dictyosteliales
49.6/96
670386.D3B7W8..Heterostelium_album_PN500 Amoebozoa..Evosea..Eumycetozoa..Dictyostelids..Acytosteliales
85.4/94
670386.D3B7W7..Heterostelium_album_PN500 Amoebozoa..Evosea..Eumycetozoa..Dictyostelids..Acytosteliales
77.5/49
71.9/85
5759.C4LU79..Entamoeba_histolytica Amoebozoa..Evosea..Archamoebae..Entamoebidae..Entamoeba
1257118.L8H342..Acanthamoeba_castellanii_str._Neff Amoebozoa..Discosea..Longamoebia..Centramoebida..Acanthamoebidae
99.3/100
5722.A2E0U0..Trichomonas_vaginalis Metamonada..Parabasalia..Trichomonadida..Trichomonadidae..Trichomonas
42.1/54
1144522.A0A1J4K8D7..Tritrichomonas_foetus Metamonada..Parabasalia..Tritrichomonadida..Tritrichomonadidae..Tritrichomonas
2903.R1E5T2..Emiliania_huxleyi Haptista..Haptophyta..Prymnesiophyceae..Isochrysidales..Noelaerhabdaceae
22.9/33
97.4/100
630700.A0A3L6KYR7..Trypanosoma_brucei_equiperdum Euglenozoa..Kinetoplastida..Trypanosomatidae..Trypanosoma..Trypanozoon
88.2/99
5693.A0A2V2V5H5..Trypanosoma_cruzi Euglenozoa..Kinetoplastida..Trypanosomatidae..Trypanosoma..Schizotrypanum
92.4/99
5664.Q4QCJ8..Leishmania_major Euglenozoa..Kinetoplastida..Trypanosomatidae..Leishmaniinae..Leishmania
97.3/98
75058.A0A0S4J166..Bodo_saltans Euglenozoa..Kinetoplastida..Bodonidae..Bodo..Bodo_domain_saltans
82.7/80
59799.S9TTD6..Angomonas_deanei Euglenozoa..Kinetoplastida..Trypanosomatidae..Strigomonadinae..Angomonas
45.5/99
448386.A0A2V3IY06..Gracilariopsis_chorda Rhodophyta..Florideophyceae..Rhodymeniophycidae..Gracilariales..Gracilariaceae
70.7/36
96.9/98
36.4/46
2769.R7QAI6..Chondrus_crispus Rhodophyta..Florideophyceae..Rhodymeniophycidae..Gigartinales..Gigartinaceae
2786.A0A1X6NIM9..Porphyra_umbilicalis Rhodophyta..Bangiophyceae..Bangiales..Bangiaceae..Porphyra
98.9/100
37360.A0A0G4IR72..Plasmodiophora_brassicae Rhizaria..Endomyxa..Plasmodiophorida..Plasmodiophoridae..Plasmodiophora
75.4/62
70186.A0A0H5RAB5..Spongospora_subterranea Rhizaria..Endomyxa..Plasmodiophorida..Plasmodiophoridae..Spongospora
461836.A0A0L0DJ13..Thecamonas_trahens_ATCC_50062 Apusozoa..Apusomonadidae..Thecamonas..Thecamonas_trahens
91.7/93
905079.L1IM98..Guillardia_theta_CCMP2712 Cryptophyta..Pyrenomonadales..Geminigeraceae..Guillardia..Guillardia_domain_theta
130081.M2X7F8..Galdieria_sulphuraria Rhodophyta..Bangiophyceae..Cyanidiales..Cyanidiaceae..Galdieria
69.4/52
441375.B6A9C6..Cryptosporidium_muris_RN66 Alveolata..Apicomplexa..Conoidasida..Coccidia..Eucoccidiorida
49.7/51
1036727.A0A024WIY0..Plasmodium_falciparum_MaliPS096_E11 Alveolata..Apicomplexa..Aconoidasida..Haemosporida..Plasmodiidae
76.8/64
98.1/45
1169540.A0A0G4EBV8..Vitrella_brassicaformis_CCMP3155 Alveolata..Colpodellida..Vitrellaceae..Vitrella..Vitrella_domain_brassicaformis
85.8/57
5802.U6KHA0..Eimeria_tenella Alveolata..Apicomplexa..Conoidasida..Coccidia..Eucoccidiorida
96.9/88
933077.A0A086PNC0..Toxoplasma_gondii_VAND Alveolata..Apicomplexa..Conoidasida..Coccidia..Eucoccidiorida
49.7/70
5802.U6KHI3..Eimeria_tenella Alveolata..Apicomplexa..Conoidasida..Coccidia..Eucoccidiorida
98.2/82
1169474.A0A0K6S7B7..Chromera_velia_CCMP2878 Alveolata..Colpodellida..Chromeraceae..Chromera..Chromera_domain_velia
99.8/100
189622.A0A2H6K8Q5..Babesia_ovata Alveolata..Apicomplexa..Aconoidasida..Piroplasmida..Babesiidae
58.4/67
5865.A7AMU5..Babesia_bovis Alveolata..Apicomplexa..Aconoidasida..Piroplasmida..Babesiidae
38.8/87
110365.A0A023BCQ0..Gregarina_niphandrodes Alveolata..Apicomplexa..Conoidasida..Gregarinasina..Eugregarinorida
46433.X6N3Q9..Reticulomyxa_filosa Rhizaria..Retaria..Foraminifera..Monothalamids..Reticulomyxidae
91.7/99
84.9/36
695850.A0A067C117..Saprolegnia_parasitica_CBS_223.65 Stramenopiles..Oomycetes..Saprolegniales..Saprolegniaceae..Saprolegnia
99.8/100
403677.D0NVN9..Phytophthora_infestans_T30-4 Stramenopiles..Oomycetes..Peronosporales..Peronosporaceae..Phytophthora
63.3/95
65357.A0A024GT08..Albugo_candida Stramenopiles..Oomycetes..Albuginales..Albuginaceae..Albugo
95/100
89.8/99
2880.D8LQM4..Ectocarpus_siliculosus Stramenopiles..PX_domain_clade..Phaeophyceae..Ectocarpales..Ectocarpaceae
1027361.A0A4D9D2G5..Nannochloropsis_salina_CCMP1776 Stramenopiles..Eustigmatophyceae..Eustigmatales..Monodopsidaceae..Microchloropsis
37.9/69
99.2/100
556484.B7G3S0..Phaeodactylum_tricornutum_CCAP_1055_1 Stramenopiles..Bacillariophyta..Bacillariophyceae..Bacillariophycidae..Naviculales
81.7/45
35128.B8C0F4..Thalassiosira_pseudonana Stramenopiles..Bacillariophyta..Coscinodiscophyceae..Thalassiosirophycidae..Thalassiosirales
2315210.A0A2R5GHB9..Hondaea_fermentalgiana Stramenopiles..Labyrinthulomycetes..Thraustochytriaceae..Hondaea..Hondaea_domain_fermentalgiana
59.9/40
12968.D8M0N6..Blastocystis_hominis Stramenopiles..Blastocystidae..Blastocystis..Blastocystis_domain_hominis
IPR040000_1313.14117___IPR040000_1313__GiNop9__giardia_intestinalis____metamonada______fornicata
100/100
284590.Q6CNC2..Kluyveromyces_lactis_NRRL_Y-1140 Opisthokonta..Fungi..Dikarya..Ascomycota..saccharomyceta
75.1/97
IPR001313_040000.P47077________IPR001313_040000________NOP9____saccharomyces_cerevisiae________fungi___opisthokonta
89.7/77
284812.Q92347..Schizosaccharomyces_pombe_972h- Opisthokonta..Fungi..Dikarya..Ascomycota..Taphrinomycotina
12.3/43
5061.CADANGAP00011079..Aspergillus_niger Opisthokonta..Fungi..Dikarya..Ascomycota..saccharomyceta
97.3/99
237631.A0A0D1CQU2..Ustilago_maydis_521 Opisthokonta..Fungi..Dikarya..Basidiomycota..Ustilaginomycotina
26.6/58
486041.B0DK79..Laccaria_bicolor_S238N-H82 Opisthokonta..Fungi..Dikarya..Basidiomycota..Agaricomycotina
100/100
73868.A0A1Y3NKA9..Piromyces_sp._E2 Opisthokonta..Fungi..Fungi_domain_incertae_sedis..Chytridiomycota..Neocallimastigomycetes
59.3/69
69.7/74
1754190.A0A1Y2D5W0..Neocallimastix_californiae Opisthokonta..Fungi..Fungi_domain_incertae_sedis..Chytridiomycota..Neocallimastigomycetes
86.7/66
403673.A0A177WYA0..Batrachochytrium_dendrobatidis_JEL423 Opisthokonta..Fungi..Fungi_domain_incertae_sedis..Chytridiomycota..Chytridiomycetes
13.4/35
578462.A0A0L0RVX6..Allomyces_macrogynus_ATCC_38327 Opisthokonta..Fungi..Fungi_domain_incertae_sedis..Blastocladiomycota..Blastocladiomycetes
280699.M1V7T7..Cyanidioschyzon_merolae_strain_10D Rhodophyta..Bangiophyceae..Cyanidiales..Cyanidiaceae..Cyanidioschyzon
26/75
595528.A0A0D2WMP6..Capsaspora_owczarzaki_ATCC_30864 Opisthokonta..Opisthokonta_domain_incertae_sedis..Ichthyosporea..Capsaspora..Capsaspora_owczarzaki
54.6/75
691883.A0A058Z9H6..Fonticula_alba Opisthokonta..Nucleariidae_domain_and_Fonticula_group..Fonticulida..Fonticulaceae..Fonticula
667725.A0A0L0FQZ9..Sphaeroforma_arctica_JP610 Opisthokonta..Opisthokonta_domain_incertae_sedis..Ichthyosporea..Ichthyophonida..Sphaeroforma
34.7/48
7668.W4YWP0..Strongylocentrotus_purpuratus Opisthokonta..Metazoa..Eumetazoa..Bilateria..Deuterostomia
98.1/45
34.1/46
37653.A0A0L8GA81..Octopus_bimaculoides Opisthokonta..Metazoa..Eumetazoa..Bilateria..Protostomia
83.8/86
7227.Q7JX95..Drosophila_melanogaster Opisthokonta..Metazoa..Eumetazoa..Bilateria..Protostomia
83.3/84
10228.B3S6N1..Trichoplax_adhaerens Opisthokonta..Metazoa..Placozoa..Trichoplacidae..Trichoplax
99.1/100
8364.B2GUM3..Xenopus_tropicalis Opisthokonta..Metazoa..Eumetazoa..Bilateria..Deuterostomia
99.3/99
96.1/45
IPR001313_040000.Q86U38________IPR001313_040000________NOP9____homo_sapiens____metazoa_opisthokonta
7955.E7EZJ0..Danio_rerio Opisthokonta..Metazoa..Eumetazoa..Bilateria..Deuterostomia
36.9/96
946362.F2UIP1..Salpingoeca_rosetta Opisthokonta..Choanoflagellata..Craspedida..Salpingoecidae..Salpingoeca
63.9/48
81824.A9USY7..Monosiga_brevicollis Opisthokonta..Choanoflagellata..Craspedida..Salpingoecidae..Monosiga
1076344.A0A146K3T1..Trepomonas_sp._PC1 Metamonada..Fornicata..Diplomonadida..Hexamitidae..Hexamitinae
NOP9 family
Bipartition Support:
aLRT/ultrafast bootstrap
2.0

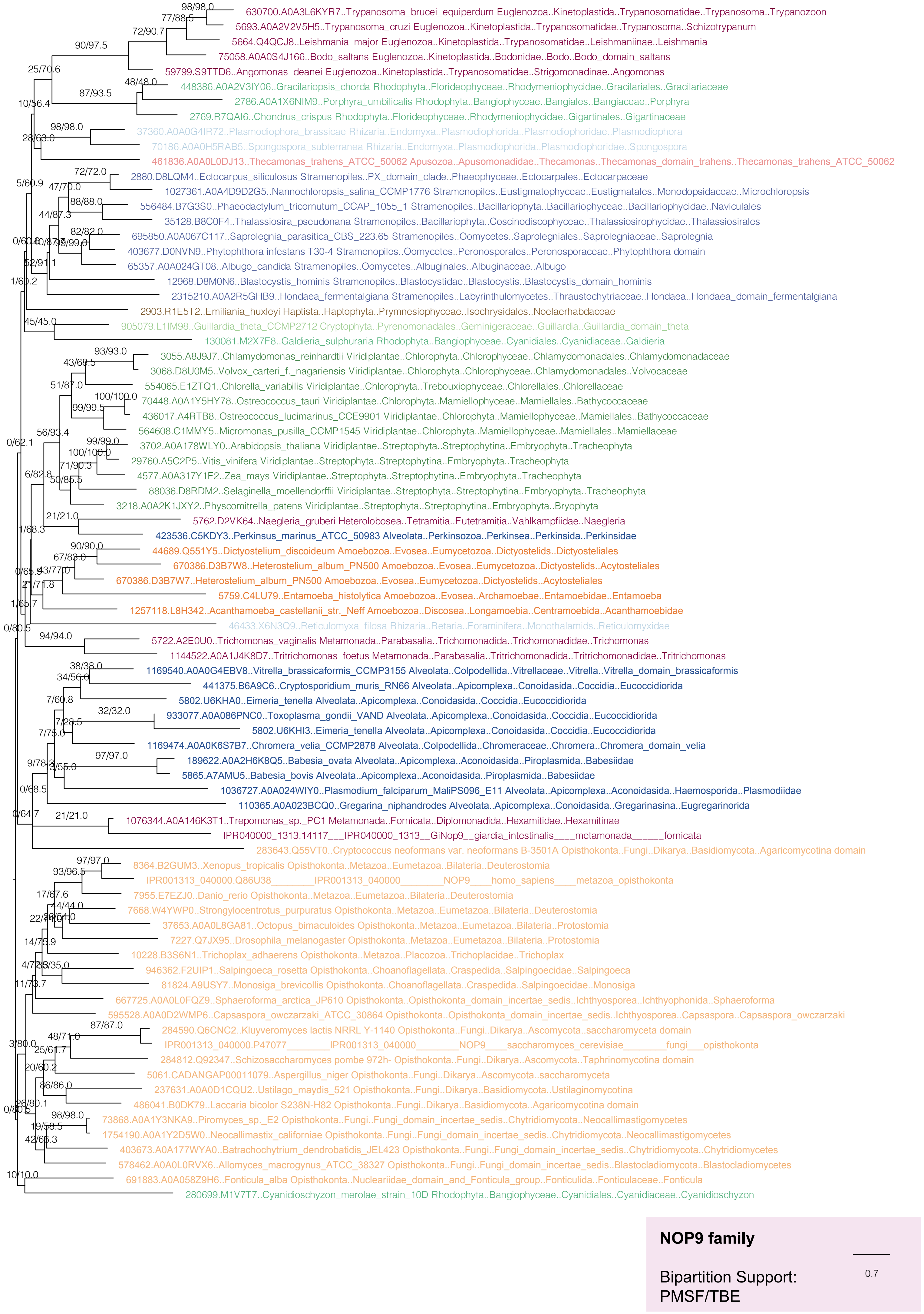
98/98.0
630700.A0A3L6KYR7..Trypanosoma_brucei_equiperdum Euglenozoa..Kinetoplastida..Trypanosomatidae..Trypanosoma..Trypanozoon
77/88.5
5693.A0A2V2V5H5..Trypanosoma_cruzi Euglenozoa..Kinetoplastida..Trypanosomatidae..Trypanosoma..Schizotrypanum
72/90.7
5664.Q4QCJ8..Leishmania_major Euglenozoa..Kinetoplastida..Trypanosomatidae..Leishmaniinae..Leishmania
90/97.5
75058.A0A0S4J166..Bodo_saltans Euglenozoa..Kinetoplastida..Bodonidae..Bodo..Bodo_domain_saltans
25/70.6
59799.S9TTD6..Angomonas_deanei Euglenozoa..Kinetoplastida..Trypanosomatidae..Strigomonadinae..Angomonas
48/48.0
448386.A0A2V3IY06..Gracilariopsis_chorda Rhodophyta..Florideophyceae..Rhodymeniophycidae..Gracilariales..Gracilariaceae
87/93.5
10/56.4
2786.A0A1X6NIM9..Porphyra_umbilicalis Rhodophyta..Bangiophyceae..Bangiales..Bangiaceae..Porphyra
2769.R7QAI6..Chondrus_crispus Rhodophyta..Florideophyceae..Rhodymeniophycidae..Gigartinales..Gigartinaceae
98/98.0
37360.A0A0G4IR72..Plasmodiophora_brassicae Rhizaria..Endomyxa..Plasmodiophorida..Plasmodiophoridae..Plasmodiophora
28/63.0
70186.A0A0H5RAB5..Spongospora_subterranea Rhizaria..Endomyxa..Plasmodiophorida..Plasmodiophoridae..Spongospora
461836.A0A0L0DJ13..Thecamonas_trahens_ATCC_50062 Apusozoa..Apusomonadidae..Thecamonas..Thecamonas_domain_trahens..Thecamonas_trahens_ATCC_50062
72/72.0
2880.D8LQM4..Ectocarpus_siliculosus Stramenopiles..PX_domain_clade..Phaeophyceae..Ectocarpales..Ectocarpaceae
5/60.9
47/70.0
1027361.A0A4D9D2G5..Nannochloropsis_salina_CCMP1776 Stramenopiles..Eustigmatophyceae..Eustigmatales..Monodopsidaceae..Microchloropsis
88/88.0
556484.B7G3S0..Phaeodactylum_tricornutum_CCAP_1055_1 Stramenopiles..Bacillariophyta..Bacillariophyceae..Bacillariophycidae..Naviculales
44/87.3
35128.B8C0F4..Thalassiosira_pseudonana Stramenopiles..Bacillariophyta..Coscinodiscophyceae..Thalassiosirophycidae..Thalassiosirales
82/82.0
695850.A0A067C117..Saprolegnia_parasitica_CBS_223.65 Stramenopiles..Oomycetes..Saprolegniales..Saprolegniaceae..Saprolegnia
0/60.6
40/87.7
99/99.0
403677.D0NVN9..Phytophthora infestans T30-4 Stramenopiles..Oomycetes..Peronosporales..Peronosporaceae..Phytophthora domain
52/91.1
65357.A0A024GT08..Albugo_candida Stramenopiles..Oomycetes..Albuginales..Albuginaceae..Albugo
1/60.2
12968.D8M0N6..Blastocystis_hominis Stramenopiles..Blastocystidae..Blastocystis..Blastocystis_domain_hominis
2315210.A0A2R5GHB9..Hondaea_fermentalgiana Stramenopiles..Labyrinthulomycetes..Thraustochytriaceae..Hondaea..Hondaea_domain_fermentalgiana
2903.R1E5T2..Emiliania_huxleyi Haptista..Haptophyta..Prymnesiophyceae..Isochrysidales..Noelaerhabdaceae
45/45.0
905079.L1IM98..Guillardia_theta_CCMP2712 Cryptophyta..Pyrenomonadales..Geminigeraceae..Guillardia..Guillardia_domain_theta
130081.M2X7F8..Galdieria_sulphuraria Rhodophyta..Bangiophyceae..Cyanidiales..Cyanidiaceae..Galdieria
93/93.0
3055.A8J9J7..Chlamydomonas_reinhardtii Viridiplantae..Chlorophyta..Chlorophyceae..Chlamydomonadales..Chlamydomonadaceae
43/68.5
3068.D8U0M5..Volvox_carteri_f._nagariensis Viridiplantae..Chlorophyta..Chlorophyceae..Chlamydomonadales..Volvocaceae
51/87.0
554065.E1ZTQ1..Chlorella_variabilis Viridiplantae..Chlorophyta..Trebouxiophyceae..Chlorellales..Chlorellaceae
100/100.0
70448.A0A1Y5HY78..Ostreococcus_tauri Viridiplantae..Chlorophyta..Mamiellophyceae..Mamiellales..Bathycoccaceae
99/99.5
436017.A4RTB8..Ostreococcus_lucimarinus_CCE9901 Viridiplantae..Chlorophyta..Mamiellophyceae..Mamiellales..Bathycoccaceae
56/93.4
564608.C1MMY5..Micromonas_pusilla_CCMP1545 Viridiplantae..Chlorophyta..Mamiellophyceae..Mamiellales..Mamiellaceae
0/62.1
99/99.0
3702.A0A178WLY0..Arabidopsis_thaliana Viridiplantae..Streptophyta..Streptophytina..Embryophyta..Tracheophyta
100/100.0
29760.A5C2P5..Vitis_vinifera Viridiplantae..Streptophyta..Streptophytina..Embryophyta..Tracheophyta
71/90.3
4577.A0A317Y1F2..Zea_mays Viridiplantae..Streptophyta..Streptophytina..Embryophyta..Tracheophyta
6/82.8
50/85.5
88036.D8RDM2..Selaginella_moellendorffii Viridiplantae..Streptophyta..Streptophytina..Embryophyta..Tracheophyta
3218.A0A2K1JXY2..Physcomitrella_patens Viridiplantae..Streptophyta..Streptophytina..Embryophyta..Bryophyta
21/21.0
5762.D2VK64..Naegleria_gruberi Heterolobosea..Tetramitia..Eutetramitia..Vahlkampfiidae..Naegleria
1/68.3
423536.C5KDY3..Perkinsus_marinus_ATCC_50983 Alveolata..Perkinsozoa..Perkinsea..Perkinsida..Perkinsidae
90/90.0
44689.Q551Y5..Dictyostelium_discoideum Amoebozoa..Evosea..Eumycetozoa..Dictyostelids..Dictyosteliales
67/83.0
670386.D3B7W8..Heterostelium_album_PN500 Amoebozoa..Evosea..Eumycetozoa..Dictyostelids..Acytosteliales
0/65.9
43/77.0
670386.D3B7W7..Heterostelium_album_PN500 Amoebozoa..Evosea..Eumycetozoa..Dictyostelids..Acytosteliales
21/71.8
5759.C4LU79..Entamoeba_histolytica Amoebozoa..Evosea..Archamoebae..Entamoebidae..Entamoeba
1/65.7
1257118.L8H342..Acanthamoeba_castellanii_str._Neff Amoebozoa..Discosea..Longamoebia..Centramoebida..Acanthamoebidae
0/80.5
46433.X6N3Q9..Reticulomyxa_filosa Rhizaria..Retaria..Foraminifera..Monothalamids..Reticulomyxidae
94/94.0
5722.A2E0U0..Trichomonas_vaginalis Metamonada..Parabasalia..Trichomonadida..Trichomonadidae..Trichomonas
1144522.A0A1J4K8D7..Tritrichomonas_foetus Metamonada..Parabasalia..Tritrichomonadida..Tritrichomonadidae..Tritrichomonas
38/38.0
1169540.A0A0G4EBV8..Vitrella_brassicaformis_CCMP3155 Alveolata..Colpodellida..Vitrellaceae..Vitrella..Vitrella_domain_brassicaformis
34/56.0
441375.B6A9C6..Cryptosporidium_muris_RN66 Alveolata..Apicomplexa..Conoidasida..Coccidia..Eucoccidiorida
7/60.8
5802.U6KHA0..Eimeria_tenella Alveolata..Apicomplexa..Conoidasida..Coccidia..Eucoccidiorida
32/32.0
933077.A0A086PNC0..Toxoplasma_gondii_VAND Alveolata..Apicomplexa..Conoidasida..Coccidia..Eucoccidiorida
7/28.5
5802.U6KHI3..Eimeria_tenella Alveolata..Apicomplexa..Conoidasida..Coccidia..Eucoccidiorida
7/75.0
1169474.A0A0K6S7B7..Chromera_velia_CCMP2878 Alveolata..Colpodellida..Chromeraceae..Chromera..Chromera_domain_velia
97/97.0
189622.A0A2H6K8Q5..Babesia_ovata Alveolata..Apicomplexa..Aconoidasida..Piroplasmida..Babesiidae
9/78.3
3/55.0
5865.A7AMU5..Babesia_bovis Alveolata..Apicomplexa..Aconoidasida..Piroplasmida..Babesiidae
1036727.A0A024WIY0..Plasmodium_falciparum_MaliPS096_E11 Alveolata..Apicomplexa..Aconoidasida..Haemosporida..Plasmodiidae
0/68.5
110365.A0A023BCQ0..Gregarina_niphandrodes Alveolata..Apicomplexa..Conoidasida..Gregarinasina..Eugregarinorida
0/64.7
21/21.0
1076344.A0A146K3T1..Trepomonas_sp._PC1 Metamonada..Fornicata..Diplomonadida..Hexamitidae..Hexamitinae
IPR040000_1313.14117___IPR040000_1313__GiNop9__giardia_intestinalis____metamonada______fornicata
283643.Q55VT0..Cryptococcus neoformans var. neoformans B-3501A Opisthokonta..Fungi..Dikarya..Basidiomycota..Agaricomycotina domain
97/97.0
8364.B2GUM3..Xenopus_tropicalis Opisthokonta..Metazoa..Eumetazoa..Bilateria..Deuterostomia
93/96.5
IPR001313_040000.Q86U38________IPR001313_040000________NOP9____homo_sapiens____metazoa_opisthokonta
17/67.6
7955.E7EZJ0..Danio_rerio Opisthokonta..Metazoa..Eumetazoa..Bilateria..Deuterostomia
44/44.0
7668.W4YWP0..Strongylocentrotus_purpuratus Opisthokonta..Metazoa..Eumetazoa..Bilateria..Deuterostomia
22/74.5
26/54.0
37653.A0A0L8GA81..Octopus_bimaculoides Opisthokonta..Metazoa..Eumetazoa..Bilateria..Protostomia
14/75.9
7227.Q7JX95..Drosophila_melanogaster Opisthokonta..Metazoa..Eumetazoa..Bilateria..Protostomia
10228.B3S6N1..Trichoplax_adhaerens Opisthokonta..Metazoa..Placozoa..Trichoplacidae..Trichoplax
4/72.5
35/35.0
946362.F2UIP1..Salpingoeca_rosetta Opisthokonta..Choanoflagellata..Craspedida..Salpingoecidae..Salpingoeca
11/73.7
81824.A9USY7..Monosiga_brevicollis Opisthokonta..Choanoflagellata..Craspedida..Salpingoecidae..Monosiga
667725.A0A0L0FQZ9..Sphaeroforma_arctica_JP610 Opisthokonta..Opisthokonta_domain_incertae_sedis..Ichthyosporea..Ichthyophonida..Sphaeroforma
595528.A0A0D2WMP6..Capsaspora_owczarzaki_ATCC_30864 Opisthokonta..Opisthokonta_domain_incertae_sedis..Ichthyosporea..Capsaspora..Capsaspora_owczarzaki
87/87.0
284590.Q6CNC2..Kluyveromyces lactis NRRL Y-1140 Opisthokonta..Fungi..Dikarya..Ascomycota..saccharomyceta domain
48/71.0
3/80.0
IPR001313_040000.P47077________IPR001313_040000________NOP9____saccharomyces_cerevisiae________fungi___opisthokonta
25/61.7
284812.Q92347..Schizosaccharomyces pombe 972h- Opisthokonta..Fungi..Dikarya..Ascomycota..Taphrinomycotina domain
20/60.2
5061.CADANGAP00011079..Aspergillus_niger Opisthokonta..Fungi..Dikarya..Ascomycota..saccharomyceta
86/86.0
237631.A0A0D1CQU2..Ustilago_maydis_521 Opisthokonta..Fungi..Dikarya..Basidiomycota..Ustilaginomycotina
26/80.1
486041.B0DK79..Laccaria bicolor S238N-H82 Opisthokonta..Fungi..Dikarya..Basidiomycota..Agaricomycotina domain
0/80.5
98/98.0
73868.A0A1Y3NKA9..Piromyces_sp._E2 Opisthokonta..Fungi..Fungi_domain_incertae_sedis..Chytridiomycota..Neocallimastigomycetes
19/58.5
1754190.A0A1Y2D5W0..Neocallimastix_californiae Opisthokonta..Fungi..Fungi_domain_incertae_sedis..Chytridiomycota..Neocallimastigomycetes
42/65.3
403673.A0A177WYA0..Batrachochytrium_dendrobatidis_JEL423 Opisthokonta..Fungi..Fungi_domain_incertae_sedis..Chytridiomycota..Chytridiomycetes
578462.A0A0L0RVX6..Allomyces_macrogynus_ATCC_38327 Opisthokonta..Fungi..Fungi_domain_incertae_sedis..Blastocladiomycota..Blastocladiomycetes
10/10.0
691883.A0A058Z9H6..Fonticula_alba Opisthokonta..Nucleariidae_domain_and_Fonticula_group..Fonticulida..Fonticulaceae..Fonticula
280699.M1V7T7..Cyanidioschyzon_merolae_strain_10D Rhodophyta..Bangiophyceae..Cyanidiales..Cyanidiaceae..Cyanidioschyzon
NOP9 family
0.7
Bipartition Support:
PMSF/TBE

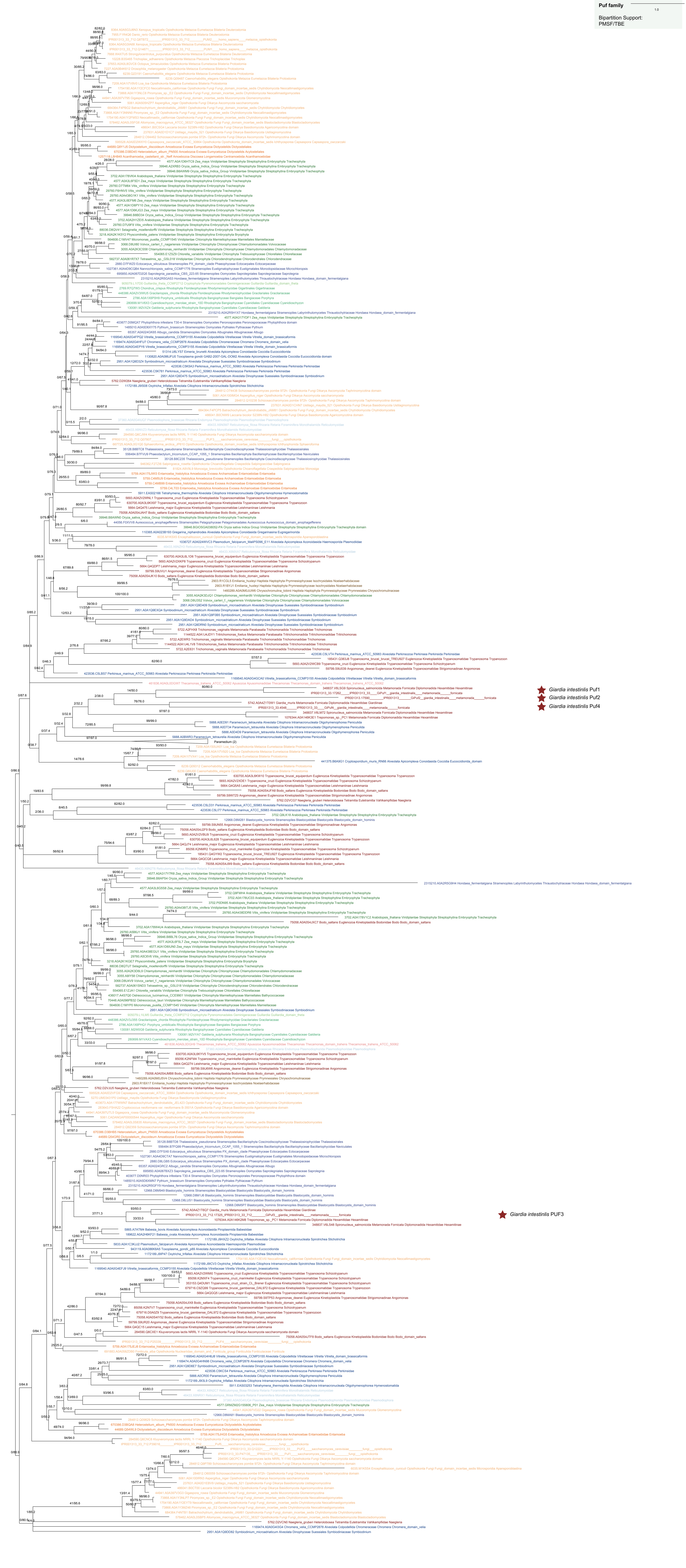
Puf family
1.0
Bipartition Support:
PMSF/TBE
Giardia intestinalis Puf1
Giardia intestinalis Puf2
Giardia intestinalis Puf4
Giardia intestinalis PUF3

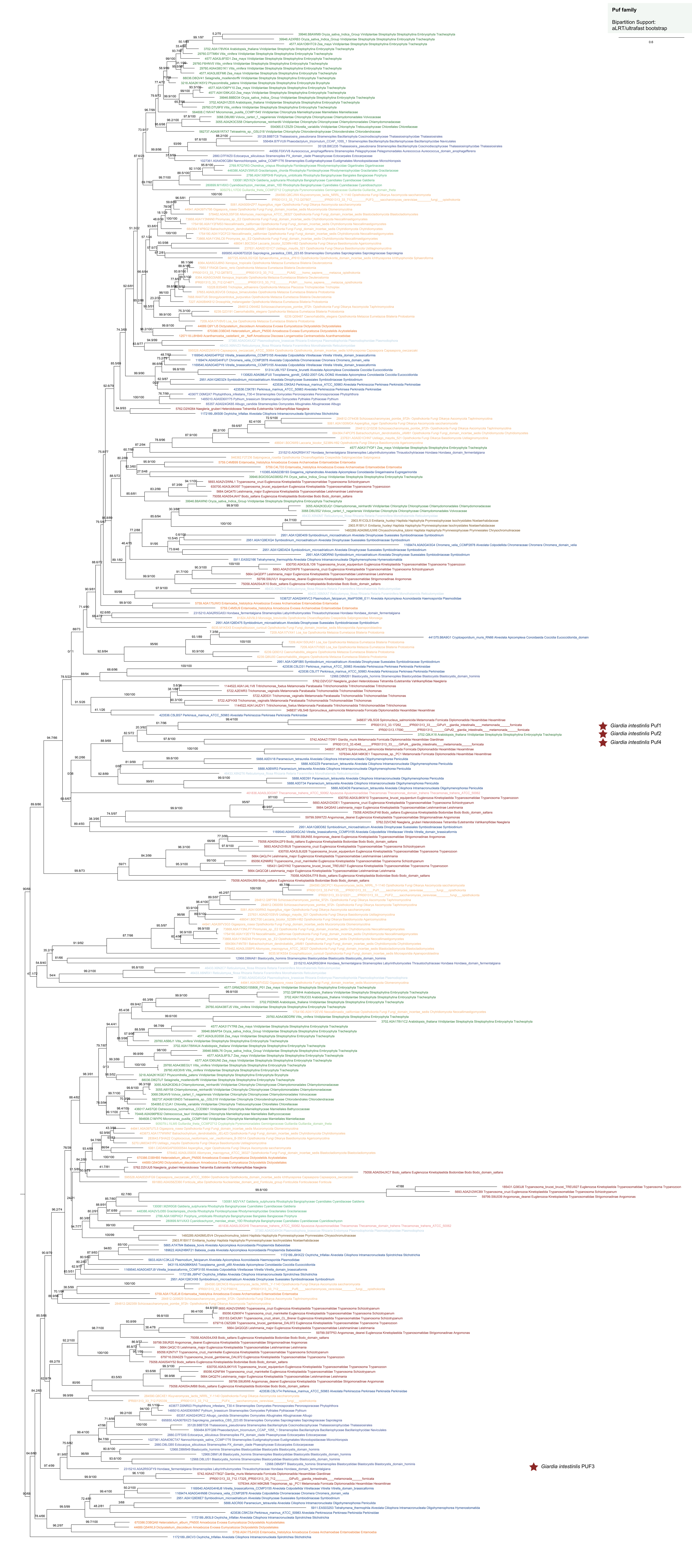
Puf family
Bipartition Support:
aLRT/ultrafast bootstrap
0.6
Giardia intestinlis Puf1
Giardia intestinlis Puf2
Giardia intestinlis Puf4
Giardia intestinlis PUF3
