# Supplementary material for: The evolution of the Puf superfamily of proteins across the tree of eukaryotes
Source: BMC Biol. 2020 Jun 30;18:77. doi: 10.1186/s12915-020-00814-3 (PMC7325665; doi:10.1186/s12915-020-00814-3)

Figure S2

```
GiNop9     1 ------------------------------------------------------------------------------MSVDLPQHVLDALDKAE-KYLCGMAS--------TSVERTEGA  34
ScNop9     1 ----------------------MGKTKTRGRRHQDKQRKDEF-EPSSNSAKEHIQQEE-----------STYNDEAEI-KETQPQMFFGVLDREELEYFKQAESTLQLDAFEAPEEKFQFV  86
AtPUM23    1 MVSVGSKSLPSRRRHRTIEEDSLMGERGKSSNNHSERNKGMRR-KDHKGNRGFDVDSSKKNQSGGAPNVKPASKKHSEF--EHQNQFVRKEIDPETSKYFSEIANLFD-SNEVELEERSVIC 117
HsNop9     1 ---------------------------MGQGPRSPHKVGRRF--PAGGKRGRGAKGSGRPLPGRKRQPWPPPDGRSEPAPDSHP-----HLSPEALGYFRRALSALK-EAPETGEERDLMV  86
DmNop9     1 -----------------------MQAEGDSRRKRPKKKGNRFMRNAKGFAKQGIFGRG------------------------------THIDDEQFNYFINILDAMK-VGFEDVEERVIMA  67

GiNop9    35 NTVFTIARGHTIPLAEHTFGARMLEEALQVGTRDQISAVFTELYPSIMTVMRSESGSHVCQTLLALI------------------SRIYR-DDCSSLSMESLGVVSAINKADGSLAELYSA 136
ScNop9    87 TSIIEEAKGKELKLVTSQITSKLMERVILECDETQLKDIFQSFNGVFFGLSCHKYASHVLETLFVR--------------------------SAALVERELLTPSFDNNEKEGPYVTMENM 181
AtPUM23  118 GNALEETRGREYEIATDYIISHVLQTLLEGCELDQLCSFIRNSASVFPAIAMDRSGSHVAESALKSL------------------ATHLENPDAYSVIEEA-------------------- 200
HsNop9    87 HNIMKEVETQALALSTNRTGSEMLQELLGFSPLKPLCRVWAALRSNLRTVACHRCGVHVLQSALLQL------------------PRLLG--SAAEEEEEE-----EEDGKDGPTETLEEL 182
DmNop9    68 NNVFEQTHDQEIHLASNQIVSKALESLVGFVDDVQLERFFSKFGDNLRPMCSDRFASHVLQKMLEIAFLRGVGKSAAQDTSDAASANKKAKPDAAQEEEEY---NLQADFTDDHREKCRQF 185

GiNop9   137 L--VLQKIIPEIPSLITHKFTNHVLRTLLRVEGGSPPED---EDRGRRKHANESTFCTMYGPTDPFAGVCSNLFIP---FSQALY-------KTLQG----------------QSTIYQSC 226
ScNop9   182 FLFMLNELKPHLKTMMNHQYASHVLRLLILILSSKTLPNS-TKANSTLRSKKSKIARKMIDIKDND-DFNKVYQTP-ESFKSELRDIITTLYKGFTNGAESRSDISQSTI----TKFREYS 295
AtPUM23  201 LHSICKVIVDNPLDMMCNCYGSHVLRRLLCLCKGVSLDSPELYGAKSSKALAKRLNLKMSQLDDNN------LEIPHQGFPGML------------------TYLLSGLLSCSREDMKYLQ 297
HsNop9   183 VLGLAAEVCDDFLVYCGDTHGSFVVRTLLQVLGGTILESERARPRGSQSSEAQKTPAQECKPAD--------FEVP-ETFLNRL------------------QDLSSSFL----KDIAVFI 272
DmNop9   186 VLRISKFMLNNLEDFVWDACASHIMRTAILCLVGMHVPKIAFEKGGTEIAKHR----KMYSVPDEW------QEVM-KEFPQRLE-------MWPQFGDFPYQDHSSSLLGV--ICLALSV 286

GiNop9   227 FDTYKSVLLQTYCKIIARCKESIDIPTVQQPLGVLLQLLNLPTTIIDKVTRKKLKDFTLKFSTFKDLCKNTSACFALESVFESVSVSQPEACGALANFVISGTTLSRTYNEFKDLSVIAGS 347
ScNop9   296 VDKVASPVIQLIIQVEGI--------------------FDRDRSFWRLV--FNTADEKDPKEE-------------------------------------------------SFLEYLLSD 345
AtPUM23  298 VDQYSSLVLQTALRLMLK----------------------QDEQLLEIIPLILRCNSTNKKVEGFHI-ETNVA---------------------------------------KEILESMKD 356
HsNop9   273 TDKISSFCLQVALQVLHR--------------------------------------------------------------------KLPQFCAHLCNAVIGYLSTRGSSVDGSPLLLFLRD 325
DmNop9   287 VDK--SMLKHFAKKILTI---------------GLLKSSDEDEEKMELDTKIEIKDEDDDNDEKEDK-KAKEADADTENKQEE-----------------------------PKLPKVFHH 360

GiNop9   348 KTASHVFSVIIGFCDDPRLLTDLLGVYTASCSVIFEHDYSKLIQSLFRGLCRAAT-FVSKTLKESSEEENVAQNDNQNKNAMDVISPELVASFEKAQRTFIDAIIELTEADKRFIRALLLP 467
ScNop9   346 PVGSHFLENVIGSARLKYVER-------------LYRLYMKDRIVKL--AKRDTTGAFVVRALLEHLKE----------KDVKQILDAVVPELSMLLNSNMDFGTAIINASNK-------- 433
AtPUM23  357 NSFSHLVEVILEVAPESLYNE-------------MFNKVFKNSLFEL--SVDRCAN-FVIQALISHARDQ---------EQMGIMWEELAPRFKDLLEQGKSGVVASLIAVSQ-------- 444
HsNop9   326 QTSSRLLEQVLLVLEPPRLQS-------------LFEEHLQGQLQTL--AAHPIAN-FPLQRLLDAVTTP---------ELLSPVFEELSPVLEAVLAQGHPGVVIALVGACR-------- 413
DmNop9   361 QSSVILLETIMSVAGAKLMIQ-------------LYAMLFCNRIGYL--ARQPGTN-FSVQRLLQHIKEV---------PDFESVFTELQPHVEELLKMGYTGVVSALSAACL-------- 448

GiNop9   468 GQPLTGPFSLEFSFSTNSKYMFKALATTFADKSHVTPEFKQQRREEHQRREKLAKEQFFG---------TKYKKKEAGTITHAGVWQTGKGIVISWFNMGNPDI-------VNMAAESLID 572
ScNop9   434 ------------QGGYLRDDVIAQLIQKY----YPEKSDAKNILESCLLLSASTL----GNTRDDWPTAEERRR---------------SVFLEQLIDYDDKFLNITIDSMLALPEERLI- 518
AtPUM23  445 ------------RLQSHENKCCEALVGAV----CSTNESRISI-----LPRLLFLDYYFGCRDKSTWEWAPG----------AKMHVMGCLILQGIFKFSSDHIQPYITSLTSMKAEYIT- 533
HsNop9   414 ------------RVGAYQAKVLQLLLEAF----HCAEPSSRQVACVPLFATLMAYEVYYGLTEEEGAVPAEHQVAMAAARALGDVTVLGSLLLQHLLHFSTPGL--VLRSLGALTGPQLL- 515
DmNop9   449 ------------RLATKQSQMIAALQSAL----HVSGDKEKSKLFLTCLIKLKPFEVVTG---------DES----------AFVHLHGSLITQHLLQFNKPIF--LVNCILDLPATQLA- 531

GiNop9   573 YSFPDPGDAFKLIMHPAGCDVYQACLGSVYVAPWYKIKLINNLKPVIDKLARNKLGCLVLTALFDATLSDTGDIPADVIEASRIVLASEIDVRA--ASASFHGRQLCAYMKLDEFQRSGED 691
ScNop9   519 ----------QMCYHGVFSHVVEHVLQTTRVDIIKRKMLLNILSKESVNLACNVYGSHIMDKLWEFTAKL---------TLYKERIARALVLETEKVKNSIYGRQVWKNWKLELYVRKMWD 620
AtPUM23  534 ----------ETAKDSSGARVIEAFLASD-AATKQKRRLIIKLRGHFGELSLHTSGSFTVEKCFDACN-----------LTLREAIASELLDVKVDLSKTKQGPYLLRKLDIDGYASRPDQ 632
HsNop9   516 ----------SLAQSPAGSHVLDAILTSPSVTRKLRRRVLQNLKGQYVALACSRHGSRVLDAIWSGAA-----------LRARKEIAAELGEQNQELIRDPFGHHVARNVALTTFLKRREA 615
DmNop9   532 ----------QVFNTPNGSHIVDAFMQSKFIGEKSRERLIRQLDGFYVDLAITRHGSRVFEQCFKAAQ-----------DAQKLRMAKELFSKANMLKGSPHGRIIYTKYRLDTYKLSPTQ 631

GiNop9   692 WSRTL--------------------QRRTEVRKEIMSIF-----------------DDAAE-------------------------------------PRRRRKA---------------- 722
ScNop9   621 WKKLIKEQEFEIFPNSKPLQPKPEKHSRERNNSKEGSAF-------------------------------------------------------------KKQKHYR-------------- 666
AtPUM23  633 WKSRQ--------------------EAKQSTYNEFCSAFGSNKSNFPKNTFVSDASEDAAQEIEVKNTRKEIDHHPTSGFKRHREKHAKDKDEPFAGEKRSKQKKNKTSEATDKPKLAGSK 733
HsNop9   616 WEQQQ--------------------GAVAKRRRALNSIL-----------------ED--------------------------------------------------------------- 636
DmNop9   632 WQESL----------SKHLDAEQTKETKRKTAKTAVEAF-----------------KDILS------------------------------------------------------------ 665
```

Figure S3

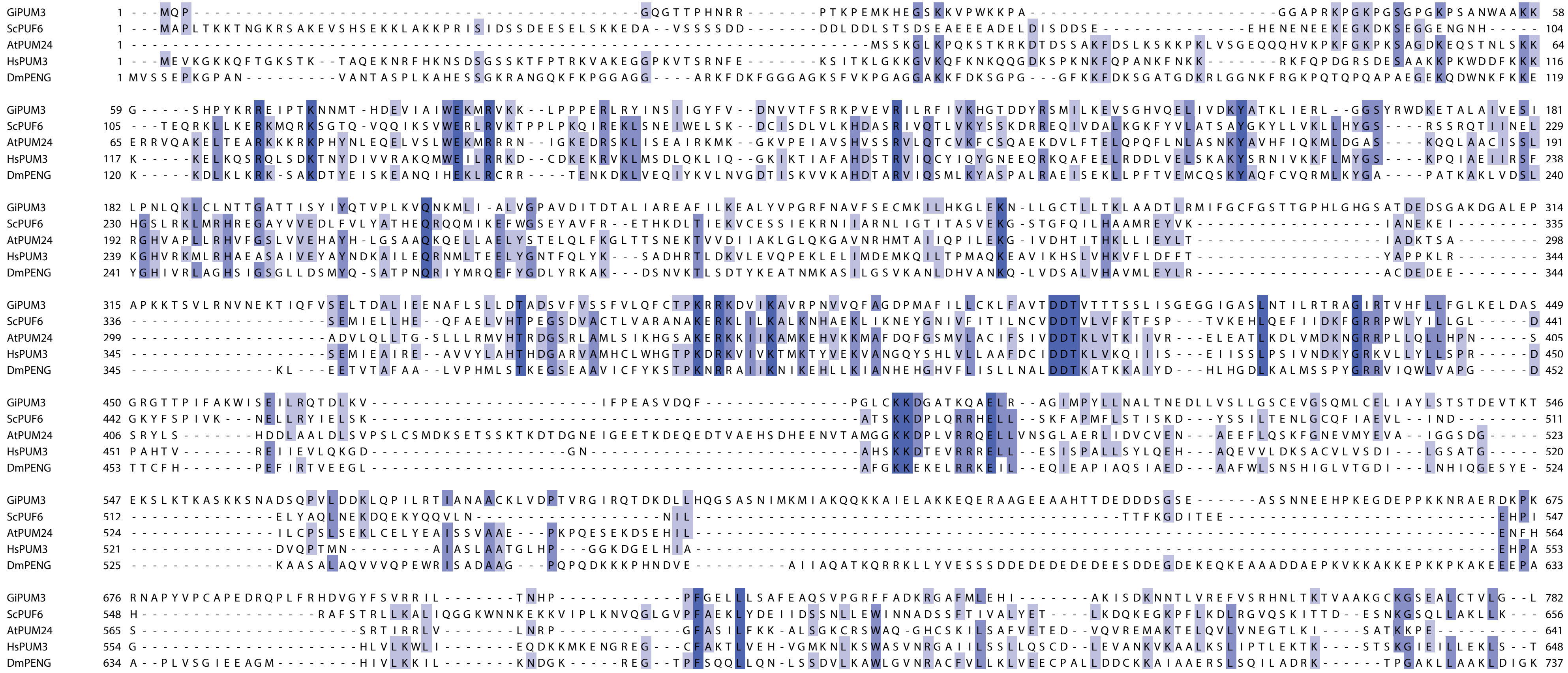

Supplement: Supplementary file 4 — Additional file 4: Figure S2. Proteins sequence alignment of Nop9 orthologues. Figure S3. Protein sequence alignment of PUM3 orthologues. [file 12915_2020_814_MOESM4_ESM.pdf]
