# Supplementary figures and images for: The evolution of the Puf superfamily of proteins across the tree of eukaryotes
Source: BMC Biol. 2020 Jun 30;18:77. doi: 10.1186/s12915-020-00814-3 (PMC7325665; doi:10.1186/s12915-020-00814-3)

Figure S4

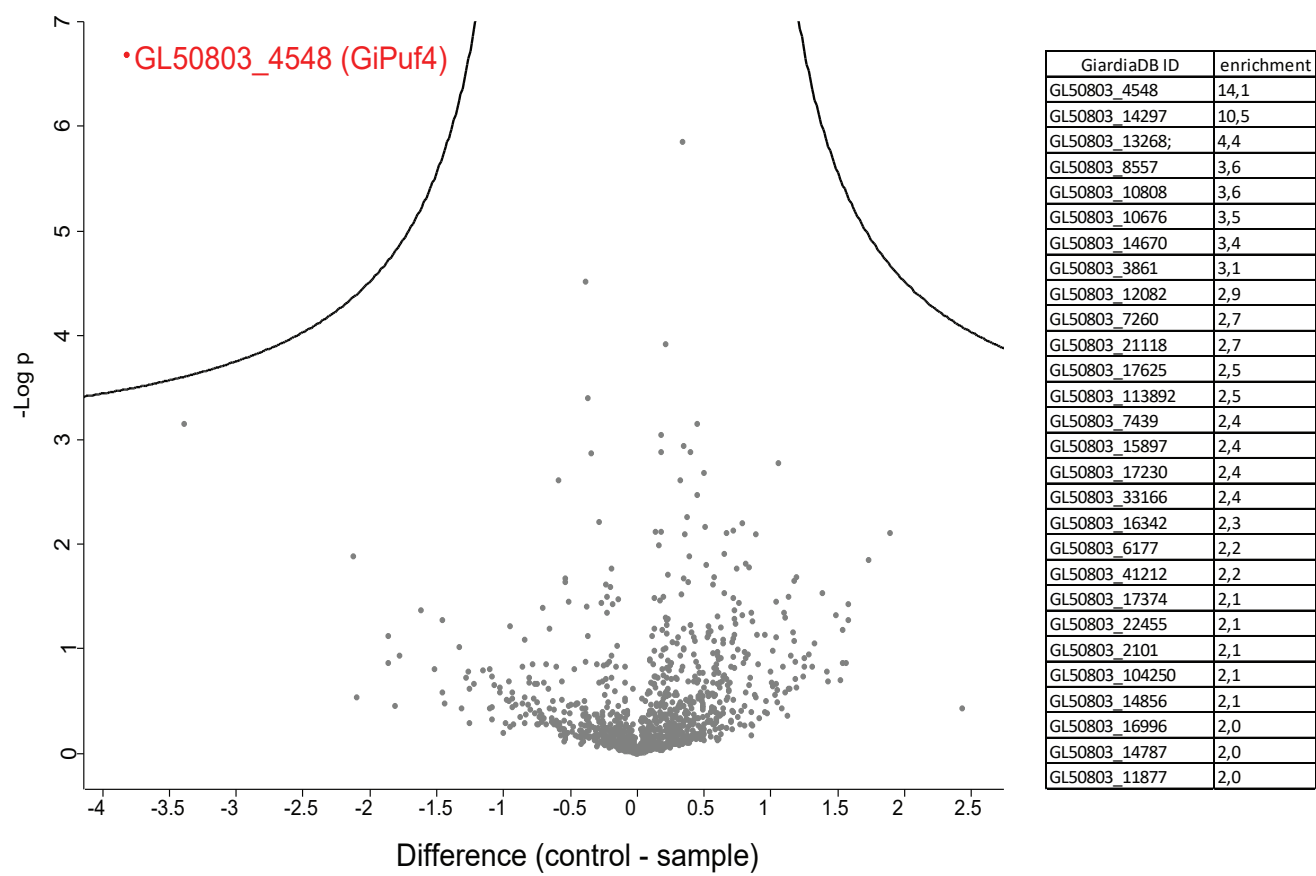

Supplement: Supplementary file 5 — Additional file 5: Figure S4. Volcano plot of proteomics analysis of GiPuf4 pull down. [file 12915_2020_814_MOESM5_ESM.pdf]
