# Supplementary material for: The evolution of the Puf superfamily of proteins across the tree of eukaryotes
Source: BMC Biol. 2020 Jun 30;18:77. doi: 10.1186/s12915-020-00814-3 (PMC7325665; doi:10.1186/s12915-020-00814-3)
Supplement: Supplementary file 9 — Additional file 9: Table S6. Primers used in the study. [file 12915_2020_814_MOESM9_ESM.docx]

Table S6

| 5´UTR-GiPuf1 ClaI F | CTAGATCGATCGAAAAGAGGGGCTTCAA |
| --- | --- |
| 5´UTR-GiPuf1 BspEI,MluI,XhoI R | CTAGCTCGAGACGCGTTCCGGATTTTTGAGGCTCTTTATA |
| 5´UTR-GiPuf2 ClaI F | CTAGATCGATCTTAGTGCAGAGCCTTAG |
| 5´UTR-GiPuf2 BspEI,MluI,XhoI R | CTAGCTCGAGACGCGTTCCGGAAGCTAGAGACTACTACCC |
| 5´UTR-GiPuf3 ClaI F | CTAGATCGATAGGTTATGGTCTGCAATC |
| 5´UTR-GiPuf3 BspEI,MluI,XhoI R | CTAGCTCGAGACGCGTTCCGGATTTAGGCTTTTATTGTCC |
| 5´UTR-GiPuf4 ClaI F | CTAGATCGATGATAGCACAGTTCAAAGT |
| 5´UTR-GiPuf4 BspEI,MluI,XhoI R | CTAGCTCGAGACGCGTTCCGGAAAAGGTGGTCCGACGACA |
| 5´UTR-GiNop9 ClaI F | CTAGATCGATAGCAGATCTGTTAGGATG |
| 5´UTR-GiNop9 BspEI,MluI,XhoI R | CTAGCTCGAGACGCGTTCCGGAGAGCAATTAAATCTAATA |
| GiPuf1 BspEI F | CTAGTCCGGAATGTTCACCGATAGTCCATCTAGCCCA |
| GiPuf1-TEV-BAP MluI R | CTAGACGCGTCTACTCGTGCCATTCTATCTTCTGAGCCTCAAAGATGTCATTTAGGCCTCCTTGAAAATACAAATTTTCACAGAAAAAGGGAAGTGCCAG |
| GiPuf2 BspEI F | CTAGTCCGGAATGGTGCTGCAACTAATGTTTTGCGTT |
| GiPuf2-TEV-BAP MluI R | CTAGACGCGTTTACTCGTGCCATTCTATCTTCTGAGCCTCAAAGATGTCATTTAGGCCTCCTTGAAAATACAAATTTTCCATAGCGTCCGAGCAGCCCAT |
| GiPuf3 BspEI F | CTAGTCCGGAATGACACTTTTACCGAAGCATTCACTG |
| GiPuf3-TEV-BAP MluI R | CTAGACGCGTTCACTCGTGCCATTCTATCTTCTGAGCCTCAAAGATGTCATTTAGGCCTCCTTGAAAATACAAATTTTCTCTGGATTTGTGTGAATTTGC |
| GiPuf4 BspEI F | CTAGTCCGGAATGAGGCATATGGACGATGCCGACAGC |
| GiPuf4 -TEV-BAP MluI R | CTAGACGCGTTTACTCGTGCCATTCTATCTTCTGAGCCTCAAAGATGTCATTTAGGCCTCCTTGAAAATACAAATTTTCTGCACAGAAAACCCACGAGCT |
| GiPUM3 NdeI F | CTAGCATATGCAGCCGGGTCAGGGGACTACCCCG |
| GiPUM3 BamHI noSTOP R | CTGAGGATCCCAGGCCCAAAACTGTGCAAAGCGC |
| GiNop9 BspEI F | CTAGTCCGGAATGTCTGTTGATCTCCCGCAGCACGTC |
| GiNop9-BAP-TEV MluI R | CTAGACGCGTCTACTCGTGCCATTCTATCTTCTGAGCCTCAAAGATGTCATTTAGGCCTCCTTGAAAATACAAATTTTCGGCCTTTCTGCGCCTTCTAGG |
